# Centennial‐scale atmospheric CO_2_ rise increased photosynthetic efficiency in a tropical tree species

**DOI:** 10.1111/nph.20358

**Published:** 2025-02-12

**Authors:** Sophie A. Zwartsenberg, Frank J. Sterck, Lenny Haddad, Jürgen Schleucher, Niels P. R. Anten, Alejandro Morales, Lucas A. Cernusak, José A. Medina‐Vega, Mizanur Rahman, Mart Vlam, Ingo Heinrich, Pieter A. Zuidema

**Affiliations:** ^1^ Forest Ecology and Forest Management Group Wageningen University & Research 6700 AA Wageningen the Netherlands; ^2^ Department of Medical Biochemistry and Biophysics Umeå University 90187 Umeå Sweden; ^3^ Centre for Crop Systems Analysis Wageningen University & Research 6700 AK Wageningen the Netherlands; ^4^ College of Science and Engineering James Cook University Cairns Qld 4878 Australia; ^5^ Forest Global Earth Observatory Smithsonian Tropical Research Institute Washington DC 20560 USA; ^6^ Department of Forestry and Environmental Science Shahjalal University of Science and Technology Sylhet 3114 Bangladesh; ^7^ Van Hall Larenstein University of Applied Sciences 6882 CT Velp the Netherlands; ^8^ DAI Deutsches Archäologisches Institut Podbielskiallee 69‐71 14195 Berlin Germany

**Keywords:** atmospheric CO_2_, canopy tree, photorespiration, photosynthesis, photosynthetic efficiency, *Toona ciliata*, tree‐ring, tropical forest

## Abstract

- Tropical forests substantially influence the terrestrial carbon sink. Their contributions to the forest carbon sink may increase due to the stimulation of photosynthesis by rising atmospheric CO_2_ (*C*_a_); however, the magnitude of this effect is poorly quantified for tropical canopy trees.
- We measured the ratio of two deuterium isotopomers of glucose derived from tree rings to estimate how photosynthetic efficiency (photorespiration‐to‐photosynthesis ratio) has responded to *C*_a_ rise at a centennial scale. Wood samples were obtained from *Toona ciliata* trees from three climatically distinct forests in Asia and Australia. We applied Bayesian mixed effect models to test how the isotopomer ratio changes with *C*_a_, tree diameter (as a proxy for crown exposure), temperature, and precipitation.
- Across all sites, long‐term *C*_a_ rise increased photosynthetic efficiency, likely due to increased photosynthesis and the concurrent suppression of photorespiration. Increasing tree size reduced photosynthetic efficiency, likely due to reduced leaf internal CO_2_ at higher irradiance and stronger hydraulic limitation. Associations of photosynthetic efficiency with temperature and precipitation were inconclusive.
- Our study reveals a centennial‐scale association between photosynthetic efficiency and increasing *C*_a_ in canopy trees and provides a new and independent line of evidence for *C*_a_‐induced stimulation of photosynthetic efficiency in tropical forests.

## Introduction

Rising atmospheric CO_2_ (*C*_a_) is rapidly changing the climate of our planet. *C*_a_ rise is expected to increase the carbon sink of terrestrial ecosystems, thereby reducing the rate of climate change. These expectations are based on the understanding that photosynthesis increases in response to elevated ambient CO_2_ levels, a phenomenon known as CO_2_ fertilization (Walker *et al*., 2020). Photosynthesis is the driver of the largest flux of carbon between the atmosphere and the biosphere (Keenan & Williams, 2018; Ryu *et al*., 2019), and the effects of CO_2_ on photosynthesis are studied from leaf to global levels. Multiple lines of empirical evidence suggest rising *C*_a_ has likely increased global terrestrial gross primary production (GPP) (Campbell *et al*., 2017; Cernusak *et al*., 2019; Walker *et al*., 2020), by as much as 13.5% between 1981 and 2020 (Keenan *et al*., 2023). Tropical forests make up a third of global terrestrial GPP (Beer *et al*., 2010) and global biomass production (Pan *et al*., 2011), and account for half of the global forest carbon sink (Bonan, 2008). Understanding the response of tropical forests to rising *C*_a_ is therefore relevant, as rising *C*_a_ may alter these contributions. Forests may be particularly responsive to *C*_a_ rise, as virtually all tree species use the C_3_ photosynthetic pathway (Sage & Sultmanis, 2016; Young *et al*., 2020) and rising *C*_a_ is expected to have the strongest effect on C_3_ photosynthesis (Lambers & Oliveira, 2019). A better understanding of *C*_a_ on canopy trees is especially important as the upper canopy layer of a forest accounts for a large fraction of total photosynthesis (Lamour *et al*., 2023). However, experimental evidence for tropical canopy tree responses to CO_2_ is still missing, as tropical forest free air CO_2_ enrichment (FACE) experiments have yet to start (Lapola & Norby, 2014; Rammig & Lapola, 2023).

**Fig. 1:**
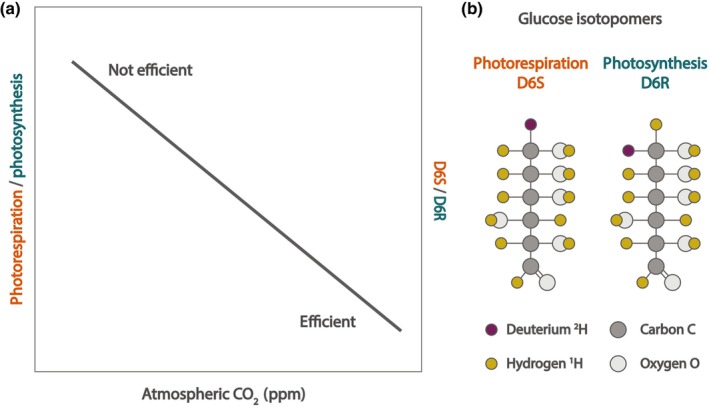
Studying long‐term *C*_a_‐induced shifts in photosynthetic efficiency (photorespiration/photosynthesis) using the ratio of D6S and D6R glucose isotopomers. Rising *C*_a_ levels decrease the photorespiration‐to‐photosynthesis ratio (a, left axis), but these are hard to quantify over long historical periods of *C*_a_ rise. Shifts in this ratio can be chemically reconstructed from glucose isotopomers: (b) variations of glucose molecules that differ in the position of deuterium (^2^H). The ratio between D6S and D6R (a, right axis) reflects the ratio between photorespiration and photosynthesis (a, left axis) and can be measured on tree rings produced by tropical forest trees to reconstruct long‐term shifts in photosynthetic efficiency under centennial‐scale *C*_a_ rise. In the photorespiration and photosynthesis pathways, deuterium is preferentially incorporated into the D6S and D6R positions of glucose, respectively, leading to a different molecular structure (b).

CO_2_ fertilization of the C_3_ pathway works through the enzyme Rubisco, which can bind CO_2_ (carboxylation), or O_2_ (oxygenation) (Lambers & Oliveira, 2019) (Supporting Information Fig. S1). Oxygenation is followed by the photorespiratory pathway, which uses energy and causes carbon loss (Busch, 2020). The ratio of the rate of oxygenation (*V*_o_) to that of carboxylation (*V*_c_) decreases with increasing CO_2_ concentration in the chloroplast (*C*_c_) (Lambers & Oliveira, 2019). As *C*_c_ increases with increasing *C*_a_, rising *C*_a_ is expected to have reduced the ratio of photorespiration to photosynthesis for tropical trees at the leaf level (Fig. 1a). So far, the scarce empirical evidence indicative of changes in photosynthesis in tropical trees comes from δ^13^C isotopes measured on tree rings. δ^13^C measurements can be used to infer changes in average leaf internal CO_2_ (*C*_i_) and tree intrinsic water use efficiency (iWUE), which is defined as the ratio of net photosynthesis to stomatal conductance of water (*A*_n_/*g*_w_) (Franks *et al*., 2013; van der Sleen *et al*., 2017). In tropical forests, *C*_i_ and iWUE are often found to increase with *C*_a_ (Peñuelas *et al*., 2011; van der Sleen *et al*., 2015, 2017). The rise in iWUE can be attributed to increased photosynthesis, decreased transpiration, or a combination of both. However, iWUE also increases with tree height, coupled with decreasing *C*_i_ (Brienen *et al*., 2017), which can be caused by increased photosynthetic drawdown of CO_2_ due to higher canopy exposure (Farquhar *et al*., 1980, 1982) or lower stomatal conductance due to hydraulic limitation (McDowell *et al*., 2011). It is thus important to take tree size into account when studying the role of *C*_a_ and climate on photosynthesis. Based on C_3_ enzyme kinetics and δ^13^C studies, tropical canopy tree photosynthesis should have increased with *C*_a_. Yet, isotopic studies do not provide direct evidence of changes in photosynthesis (independent of water processes) with long‐term rising *C*_a_ in tropical canopy trees.

Next to *C*_a_, climatic growing conditions are also expected to influence the ratio between photosynthesis and photorespiration. High leaf temperatures are known to decrease photosynthetic efficiency due to a faster decrease in the solubility of CO_2_ vs O_2_, and a decline in Rubisco specificity leading to higher rates of *V*_o_ compared to *V*_c_ (Brooks & Farquhar, 1985). These biochemical changes lead to a proportional increase in photorespiration at high temperatures (Lambers & Oliveira, 2019). Leaf temperatures of tropical forest trees are typically high (Doughty *et al*., 2023), likely leading to the high rates of photorespiration as observed in tropical forests (Doughty, 2011), and increased energy partitioning to photorespiration with leaf warming (Pons & Welschen, 2003). Precipitation is expected to influence photosynthetic efficiency directly through its positive association with *C*_i_ (Brienen *et al*., 2011), or indirectly as stomatal closure decreases leaf cooling via transpiration, thereby increasing leaf temperatures (Doughty *et al*., 2023).

We studied the effects of centennial‐scale *C*_a_ rise and climate on tropical tree photosynthetic efficiency (the photorespiration‐to‐photosynthesis ratio) based on tree rings in three tropical forests using the isotopomer ratio D6^S^ : D6^R^, hence referred to as D6SR. D6SR is the ratio between two isotopomers of glucose that differ in the position of a deuterium on the sixth carbon (Fig. 1b). The shift in positions is related to the different enzymes that construct an intermediary of glucose in the photosynthesis and photorespiration pathways (Ehlers *et al*., 2015). D6^S^ is produced during photorespiration and D6^R^ during photosynthesis (Fig. 1b). The D6SR ratio thus directly reflects the relative rates of photorespiration and photosynthesis as they occur in the leaf. We refer to the D6SR ratio as ‘photosynthetic efficiency’, as the changes in photosynthesis vs photorespiration cannot be separated. Note that a higher photosynthetic efficiency corresponds to a lower value of the D6SR ratio (Fig. 1a). The photosynthetic efficiency derived from D6SR has been shown to increase in response to CO_2_ in multiple crops and sphagnum moss under experimental and historical *C*_a_ rise (Ehlers *et al*., 2015; Serk *et al*., 2021a,b). However, the potential to study historical changes in the ratio between photosynthesis and photorespiration has never been applied to reconstruct the effects of long‐term *C*_a_ rise on trees.

**Fig. 2:**
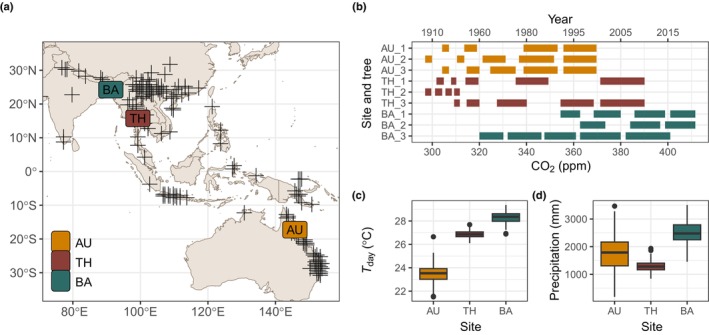
Sampling design. (a) The distribution of registered observations of *Toona ciliata* (crosses; GBIF.org, 2022) and the three sampling locations. (b) The calendar years (upper *x*‐axis) and *C*_a_ (lower *x*‐axis) values included in all samples and all sites. Our sampling design aimed to obtain wood at equidistant *C*_a_, if possible. The differing lengths of the bars are due to differences in the rate of *C*_a_ increase (i.e. short bars before 1960 and longer bars after 1960). For all samples, 10 yr were pooled except for Bangladesh, where we pooled 7 yr to obtain the minimum of three samples from younger trees. (c, d) Annual growing season temperature (*T*_day_), and growing season precipitation for all sites. For boxplots, the central line represents the median, the edges of the boxes indicate the 25^th^ and 75^th^ percentiles, and the whiskers extend to the 5^th^ and 95^th^ percentiles. Outliers, if present, are shown as individual points.

Using *Toona ciliata* M. Roem (Meliaceae), an Australasian tropical tree species, we test three hypotheses: (1) *C*_a_ has increased photosynthetic efficiency through its positive effects on *C*_i_ (decreasing the ratio of photorespiration to photosynthesis), evidenced by a decrease in the D6SR ratio (Fig. 1a); (2) photosynthetic efficiency decreases with stem diameter, a proxy for tree height and canopy light exposure, reflected by an increasing D6SR ratio; and (3) photosynthetic efficiency decreases with temperature and increases with precipitation, related to an increase and a decrease in D6SR, respectively. Additionally, we use our results to simulate the combined effects of diameter and *C*_a_ rise on photosynthetic efficiency. These simulations illustrate how photosynthetic efficiency may have changed in tropical trees growing under increasing *C*_a_ levels over the past century. This study adds an independent line of evidence to the ongoing discussion on the effects of *C*_a_ rise on tree physiology and is the first to apply the D6SR‐based reconstruction of photosynthetic efficiency to study the effects of centennial *C*_a_ rise in trees.

## Materials and Methods

### Study sites and study species description

We tested our hypotheses using tree‐ring samples of *Toona ciliata* M. Roem (Meliaceae), a deciduous tree species distributed in seasonal forests from subtropical China to subtropical Australia (Fig. 2a). *Toona ciliata* typically has a leafless period ranging from several weeks (Rahman *et al*., 2017) to several months in the dry season (Heinrich & Banks, 2005; Vlam *et al*., 2014). *Toona ciliata* is classified as a shade‐intolerant, long‐lived pioneer tree (Herwitz *et al*., 1998), reaching ages of over 180 yr (Zuidema *et al*., 2020). The species produces growth rings that can be reliably dated (Heinrich *et al*., 2008, 2009; Vlam *et al*., 2014; Rahman *et al*., 2017; Sharma *et al*., 2022), making it suitable to evaluate changes in physiology in response to climate and *C*_a_ across long periods.

Tree‐ring samples were obtained from three forests with contrasting climates: the Atherton tablelands (AU, Australia), Huai Kha Khaeng Wildlife Sanctuary (TH, Thailand), and Rema Kalenga Wildlife Sanctuary (BA, Bangladesh, Fig. 2). The Australian forest is classified as semi‐deciduous notophyll vine forest (Webb, 1959), growing on nutrient‐rich basalt soils (Brasell *et al*., 1980), with a 6‐month dry season from April to November (Heinrich *et al*., 2008). The forest in Thailand is classified as a seasonal dry evergreen and mixed deciduous forest, growing on weathered and slightly acidic ultisols (Bunyavejchewin *et al*., 2009). This site experiences a 6‐month dry season from November to April (Vlam *et al*., 2014). The site in Bangladesh is classified as a tropical semi‐evergreen forest, with slightly acidic soils (Rahman *et al*., 2017), with a 4‐month dry season lasting from November to February. The sampled forest stands in Bangladesh are patchier and, compared with the other sites, relatively disturbed, which may affect the canopy exposure of trees included in this study.

### Sampling design

D6SR isotopomer ratios were measured on stem wood taken from 5 mm increment cores, collected in 2000 for Australia, in 2014 for Thailand, and in 2016 and 2021 for Bangladesh (Fig. 2b). Three individual trees were selected for each site. Preference was given to trees that fell into the middle range of the growth‐rate distribution (within each site) and that had no major growth suppressions. We did so because long‐lasting growth suppression may indicate non climate‐related growth (and photosynthesis) variations, and the average growth rate was chosen to reduce among‐tree variation and to increase the common signal. Wood samples from the cores were taken as a time series, with a minimum of three and a maximum of five samples along the life of the tree (Fig. 2b). The number of samples per tree varied between three and five depending on the age of the tree (Fig. 2b) and covered 25–95 yr per tree. The number of samples per site was 14 for Australia, 14 for Thailand, and 12 for Bangladesh, totalling 40 samples, taken from nine trees (three per site). These samples covered a range of 20–114 cm in DBH and 298–405 ppm in *C*_a_ (Fig. S2). Each sample was re‐measured five to six times, as NMR measurements have high uncertainty, yielding 223 observations of the D6SR ratio. Thus, our data have a nested structure of observations (i.e. NMR re‐measurements) within samples (i.e. wood of different ages) within trees and within sites.

The deuterium NMR analysis used to quantify D6SR ideally uses a minimum of 0.5 g of raw material, although analysis is possible on smaller samples (Betson *et al*., 2006). To maximize the amount of wood, we pooled 10 yr of growth from one or several cores per tree. The number of years between samples in a time series was chosen to approximate equidistant *C*_a_ levels (Fig. 2b), as *C*_a_ is the variable of interest. The first sample in a time series was taken so that the mean tree diameter was at least 20 cm to ensure our reconstruction was focused on canopy trees (i.e. trees with a diameter > 27 cm were considered canopy trees by van der Sleen *et al*., 2015) and to reduce ontogenetic effects (Francey & Farquhar, 1982). The diameter at breast height (DBH) of trees during the time of ring formation was reconstructed from the tree‐ring widths and DBH field measurements (Thailand) or by combining cumulative ring width and estimates of the distance of the oldest ring to the pith (Australia and Bangladesh).

### Sample preparation for NMR isotopomer measurements

Wood samples were ground into a powder using a ball mill, and cellulose was hydrolysed into glucose following the method described by Saeman *et al*. (1945) with modifications introduced by Betson *et al*. (2006) and Schleucher *et al*. (1999) to improve yield and reproducibility. Glucose was transformed into a glucose derivative suitable for deuterium NMR measurements following Betson *et al*. (2006) (Fig. S3, details in Methods S1).

Deuterium NMR spectra were measured using an AVANCE III 850 spectrometer (Bruker) equipped with a cryogenic probe optimized for deuterium detection and equipped with a ^19^F lock. Deuterium NMR spectra were integrated by deconvolution with a Lorentzian line shape, using the TopSpin software (v.3.6.4; Bruker). The D6SR isotopomer ratio was determined as the ratio of the integrals of the D6^S^ and D6^R^ signals (Fig. S4). For each sample, five or six replicate spectra were recorded.

### Climate data

We made use of gridded climate data produced by the Climatic Research Unit (CRU v.4.07) at the 0.5‐degree resolution (Harris *et al*., 2020) for *T*_min_, *T*_max_, and precipitation, to define climatic covariates for each sample in all sites. *C*_a_ data were based on ice core data for the period 1900–1958 (Etheridge *et al*., 1996), and from the Mauna Loa observatory for observations from 1959 onwards (Keeling, 2023; Lan, 2023). All monthly climate data were subset to only include the growing season, here defined as all months with > 100 mm precipitation (Malhi *et al*., 2002), thereby allowing the length of the growing season to vary from year to year. By setting this threshold, we exclude dry months from the climate data, as *T. ciliata* is known to avoid the dry season with a leafless period. Leafless periods vary in length across sites, depending on climatology (Heinrich & Banks, 2005; Vlam *et al*., 2014; Rahman *et al*., 2017). For the Australian site, the year was considered to start in July, as a tree ring starts forming around that time of the year and continues the growth into the next calendar year (following the Schulman convention; Schulman, 1956). Growing season precipitation (Fig. 2d) was calculated as the sum of precipitation of all months with > 100 mm precipitation per year. As the processes of photosynthesis and photorespiration, underlying D6SR isotopomer ratios, happen during the daytime, we calculated the growing season day temperature (*T*_day_) defined as:

(Eqn 1) $$T_{\mathrm{day}}=\frac{1}{3}\cdot T_{\min}+\frac{2}{3}\cdot T_{\max}$$

Climate and *C*_a_ averages for each pooled sample (10 yr) were calculated as a weighted mean based on tree‐ring widths of the included years. We chose to use a weighted mean as the relative width of each included ring will change the contribution of this ring to the entire sample in absolute weight of the wood, and thus to the D6SR ratio of the sample. We calculated the relative contribution of each ring width to the total sample width, multiplied this by the annual covariate, and summed the outcome. By doing so, a wider ring contributes more to the multi‐year average of a covariate, as this ring will also contribute more to the D6SR signal.

### Model description and statistical inference

Trees experience large changes in microclimate as they grow from saplings into adults, including increased irradiance (Montgomery & Chazdon, 2001; Monsi & Saeki, 2005; Poorter *et al*., 2005; Brienen *et al*., 2022), increased air and leaf temperature (Hinckley *et al*., 2011), and increased hydraulic demands (Barnard & Ryan, 2003; Koyama *et al*., 2021; Fernández‐de‐Uña *et al*., 2023). All of these microclimatic changes are expected to level off once the tree reaches full canopy exposure, and the effects of tree diameter were therefore expected to be asymptotic, similar to trends in iWUE (due to decreasing *C*_i_) with tree diameter (McDowell *et al*., 2011; Brienen *et al*., 2017). To model the potential asymptotic effects of diameter (Hypothesis 2) on D6SR, we implemented a generalized Michaelis–Menten function (gMM) (see Martínez Cano *et al*., 2019), earlier described by Kepner (2010) for any system with saturating behaviour:

(Eqn 2) $$\begin{array}{c} D6\mathrm{SR}=\frac{a\cdot \text{diameter}}{b+\text{diameter}}\; \end{array}$$

where *a*, the asymptote, is the D6SR ratio a tree attains when it is fully exposed (large diameter, in the absence of any other variation) and *b*, the half‐saturation parameter, is the diameter at which the D6SR ratio is halfway to the asymptote. Following Hypotheses 1 and 3, we assessed how changes in *C*_a_, *T*_day_, and precipitation were associated with the maximum D6SR ratio (*a*) by allowing the asymptote to vary with these covariates. The model included a nested random effect structure on the intercept for both *a* and *b* to control for repeated observations within sites (multiple trees per site), trees (multiple measurements per individual), and samples (multiple re‐measurements of the same sample) (Details in Eqns S1.1–S1.3, Methods S2). This random effect structure effectively controls for pseudo‐replication (i.e. multiple re‐measurements per sample, tree, and site) while preserving variability within each level. Nonetheless, given that averaging re‐measurements of samples is common practice in isotope research, (Ehlers *et al*., 2015), we also fit all models using the mean value of observations per sample (Table 1). Before inference, covariates were standardized by subtracting each vector by its mean and then dividing the result by the standard deviation of the vector. Collinearity between included explanatory variables was assessed for all models and found to be low with all VIF scores under three. As the shape of the diameter effects on D6SR is based on assumption (i.e. a saturating D6SR with tree size), we assessed the robustness of the applied statistical method by comparing the results of the gMM with a linear and log‐linear shape of the diameter effect (Methods S2, Eqns S2, S3). Model performance was evaluated and compared using WAIC, LOOIC, and *R* ^2^. Model performance was similar for all tested models (Table S1), and we, therefore, present our results based on the gMM model, as the potential asymptotic effects of diameter may provide a better representation of the biology of the study system. The estimates in the text are based on the nonaveraged data, as parameter estimates from averaged data are calculated by ignoring sample‐level variability, and exhibit inflated certainty. Estimates for alternative models and models based on sample means are presented in Table 1 and Tables S2, S3.

**Table 1:** Estimates of the fixed effects for the generalized mixed Michaelis–Menten model (Eqns S1.1–S1.3), and for the linear and log‐linear mixed models (Eqns S2–S4).

Parameter	Michaelis–Menten (full)	Michaelis–Menten (means)	Linear (full)	Linear (means)	Log‐linear (full)	Log‐linear (means)
Diameter asymptote	1.016 (0.908, 1.109)	1.012 (0.899, 1.101)	–	–	–	–
Diameter half‐saturation	0.890 (0.224, 2.144)	0.736 (0.204, 1.835)	–	–	–	–
General intercept	–	–	0.999 (0.767, 1.185)	0.998 (0.808, 1.160)	0.999 (0.835, 1.135)	0.999 (0.811, 1.146)
Diameter	–	–	0.008 (−0.002, 0.019) *94%*	0.006 (0.000, 0.012) *98%*	–	–
Log diameter	–	–	–	–	0.007 (−0.001, 0.016) *96%*	0.006 (0.001, 0.011) *99%*
*C*_a_	−0.009 (−0.017, −0.001) *99%*	−0.008 (−0.013, −0.003) *100%*	−0.009 (−0.020, 0.001) *96%*	−0.009 (−0.016, −0.002) *99%*	−0.009 (−0.018, 0.000) *97%*	−0.008 (−0.014, −0.003) *100%*
*T*_day_	0.013 (−0.019, 0.069) *83%*	0.017 (−0.006, 0.052) *92%*	0.015 (−0.018, 0.072) *83%*	0.020 (−0.005, 0.057) *93%*	0.011 (−0.019, 0.067) *79%*	0.018 (−0.005, 0.054) *93%*
Precipitation	0.003 (−0.007, 0.014) *71%*	0.004 (−0.002, 0.011) *91%*	0.001 (−0.009, 0.012) *58%*	0.002 (−0.005, 0.008) *69%*	0.001 (−0.009, 0.012) *59%*	0.002 (−0.004, 0.008) *72%*

All models were fitted on both the full dataset (full), which includes all NMR re‐measurements, and on preaveraged D6SR values (means). Estimates represent the median of the posterior distribution, with the 95% credible intervals in parentheses (computed using quantiles), and the posterior probabilities (PP) of the estimate in italics. The PP reflects the probability of the true effect being positive or negative, following the sign of the estimate, given the model and data. PPs are only provided for slope estimates, as comparison with zero is not meaningful for intercepts. Details on the estimates for random effects can be found in Supporting Information Tables S2 and S3.

We fitted the model in Stan (Carpenter *et al*., 2017), with its interface in R (v.4.3.1; R Core Team, 2023) via the packages brms (v.2.20.4; Bürkner, 2017) and RStan (v.2.32.6; Stan Development Team, 2024). We incorporated weakly informative priors for both fixed and random effects (Methods S2; Fig. S5). The model coefficients were estimated using four Markov chains with 4000 iterations each, and the first 2000 were discarded as a warm‐up. Parameter convergence and Markov chain mixing were checked graphically using trace plots (Fig. S6) and numerically using Rhat values (Gelman *et al*., 2013). Goodness‐of‐fit was assessed using posterior predictive model checks (Conn *et al*., 2018; Gabry *et al*., 2019), which compares observed data to simulated predictions (Fig. S7). We report the median of posterior parameter estimates with 95% credible intervals (CIs) computed using quantiles based on the easystats package (v.0.7.0; Lüdecke *et al*., 2022). For fixed effects, we also report the posterior probability (PP) using Bayesian hypothesis testing in the easystats package (v.0.7.0; Lüdecke *et al*., 2022). The PP reflects the probability of the true effect being positive or negative, following the sign of the estimate, given the model and data. PPs are only provided for slope estimates, as comparison with zero is not meaningful for intercepts. We consider covariates to be consistently associated with the response variable (i.e. D6SR) if the CIs exclude zero, or if the PP is higher than 95%.

## Results

### Atmospheric CO_2_ increases photosynthetic efficiency

**Fig. 3:**
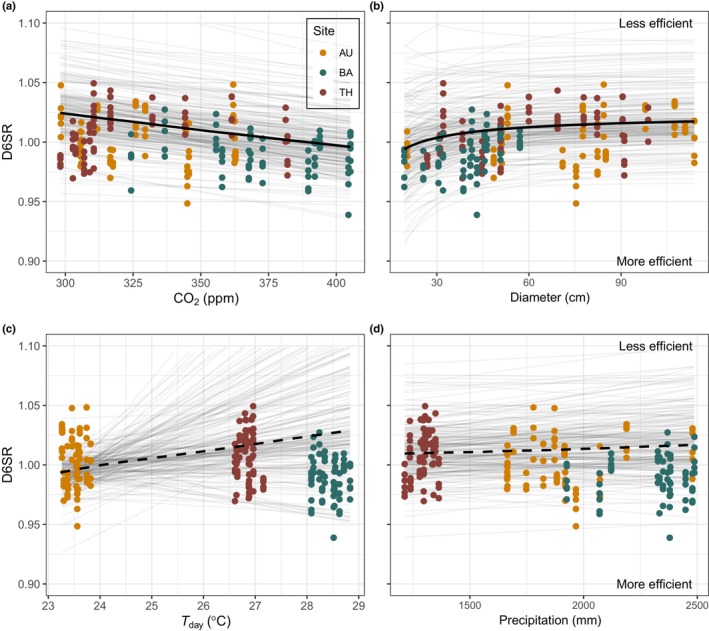
Predicted effects of atmospheric CO_2_ (*C*_a_) (a), diameter (crown exposure, b), and climate (c, d) on the photorespiration‐to‐photosynthesis ratio (D6SR) for *Toona ciliata* from three sampling sites (colours). Thick continuous lines indicate the predicted mean of the consistent associations (credible interval (CI) excludes zero and posterior probability (PP) > 95%), thick dashed lines are the predicted mean of effects that are not consistently associated (CI includes zero and PP < 95%). Thin lines represent 200 draws from the posterior predictive distributions. Predictions are based on the gMM model, inferred on the full dataset (Michaelis–Menten (*full*), Table 1), predictions for the gMM model estimated on sample means are available in Supporting Information Fig. S9.

Photosynthetic efficiency increased (i.e. a decrease in the photorespiration‐to‐photosynthesis ratio) with increasing *C*_a_ over the last 110 yr and this association was consistently observed across the three climatically distinct sites (Table S4; Fig. S8). *C*_a_ increased by 125 ppm (*c*. 36%) in the covered study period (1905–2016). This increase in *C*_a_ was negatively associated with D6SR with a slope of −0.009 (−0.017, −0.001) and a PP of 99% indicating a high probability that the slope is negative (Table 1; Fig. 3a). We find the same slope for *C*_a_ using the linear and log‐linear models with a PP of 96% and 97%, respectively (Table 1). PP values for inference based on sample means are higher for all models, indicating that estimate uncertainty is mostly rooted in NMR measurement variability (Table 1). Results are thus robust to assumptions on the shape of the diameter effect and to the inclusion of re‐measurement or preaveraging (Table 1; Fig. S9). These results support Hypothesis 1 that the ratio of photorespiration to photosynthesis decreases with increasing *C*_a_ (see Fig. S10 for the *C*_a_ association with the 1/*C*_a_ axis, as done for earlier D6SR studies). The association between the climatic variables (i.e. precipitation and day temperature) and the D6SR asymptote was inconclusive. We found no consistent association of D6SR with precipitation, as revealed by a slope of +0.003 (−0.007, +0.014) and a PP of 71% (Fig. 3C), nor with *T*_day_, as shown by a slope of +0.013 (−0.019, +0.069) and a PP of 83% (Fig. 3d). Climate associations were also inconsistent for alternative models and models based on means (Table 1).

### Crown exposure decreases photosynthetic efficiency

We found that increasing tree size is associated with a reduction in photosynthetic efficiency as demonstrated by the increase in D6SR with diameter, supporting Hypothesis 2 (Fig. 3b). Based on the gMM model, the positive association between diameter and D6SR saturates around a DBH of 60 cm and reaches an asymptote of 1.016 (+0.908, +1.109), with a half‐saturation parameter of 0.89 (+0.224, +2.144), PP values for these estimates are not given, as the relationship is based on two parameters for which comparison with zero is not informative. The mean DBH at which trees reached the asymptote coincides with the diameter at which *T. ciliata* reaches its maximum height (Fig. S11A) and holds a dominant canopy position with fully exposed crowns (Fig. S11B). For the alternative linear and log‐linear models, we found positive associations of D6SR with diameter (PP: 94% and 96%) (Fig. S12; Table 1), with higher confidence for the models based on sample means (PP: 98% and PP: 99%). Across the three tested models, LOOIC and WAIC scores were similar, and explained *c*. 64% of the variation in the data, with fixed effects explaining *c*. 33% (Table S1).

### Combined effects of *C*_a_ and tree size

**Fig. 4:**
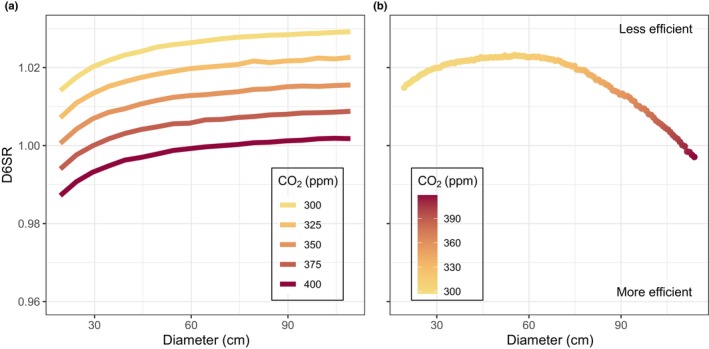
Predictions of joint effects of diameter and *C*_a_ on the photorespiration‐to‐photosynthesis ratio (D6SR) in *T. ciliata.* Simulations were done using the gMM model presented in this study under (a) different constant *C*_a_ levels using a stepwise increase from 300 ppm (year) to 400 ppm (year), and (b) the effects of a simultaneous (constant) increase in diameter and 120 yr of observed historic *C*_a_ rise (based on *C*_a_ data). These same simulations are also presented based on the alternative linear and log‐linear model in Supporting Information Fig. S13, both of which show similar patterns for the joint effects of *C*_a_ and diameter.

The association between diameter and *C*_a_ on D6SR work in opposite directions, complicating the interpretation of *C*_a_ effects. To illustrate this, we explored the joint effects of diameter and *C*_a_ using simulations from the gMM model (Fig. 4), and the alternative linear and log‐linear models (Fig. S13). Under different constant levels of *C*_a_, the D6SR asymptote shifts down with higher *C*_a_ levels, consistent with an increase in photosynthetic efficiency (Fig. 4a). If we allow diameter and *C*_a_ to increase concurrently, to mimic the development of trees under increasing *C*_a_, D6SR increases with diameter but no longer reaches an asymptote (Fig. 4b). In this scenario, a maximum value of D6SR is reached at *c*. 60 cm DBH, corresponding to the maximum decrease in photosynthetic efficiency. After reaching a maximum, D6SR decreases again, implying improved photosynthetic efficiency with rising *C*_a_, without a compensating effect of further diameter increase. The alternative linear and log‐linear models yield a similar pattern for the combined effects of diameter and *C*_a_ (Fig. S13).

## Discussion

This study used D6SR isotopomers to reconstruct historical changes in the ratio of photorespiration to photosynthesis of tropical forest canopy trees in response to rising *C*_a_, tree size, and climate. D6SR isotopomers are a relatively new method (Ehlers *et al*., 2015; Serk *et al*., 2021a,b), and this is the first study using D6SR to assess photosynthetic responses to *C*_a_ for trees. This study adds an independent line of empirical evidence demonstrating changes in tree physiology in response to increasing *C*_a_. We found that *C*_a_ increases photosynthetic efficiency, reflected by a negative association of D6SR with *C*_a_, while tree diameter, as a proxy for canopy exposure, decreased photosynthetic efficiency.

### Methodological limitations

This study marks the first application of D6SR measurements on trees, and as such, we evaluate the main methodological limitations. The D6SR measurements presented in this study exhibit relatively high variability compared with the effect sizes, primarily due to the inherent uncertainty of deuterium NMR measurements, which typically range within a few percent (Akoka & Remaud, 2020). A high measurement uncertainty can reduce the certainty of the parameter estimates, as shown by the stronger PPs and narrower CIs when using sample means compared with using all observations for inference (Table 1). Uncertainty in NMR measurements could potentially be reduced by using a larger amount of wood for the derivatives, which could be achieved by taking thicker increment cores. To account for the uncertainty of the D6SR estimate, a minimum of five re‐measurements were conducted. In addition to measurement error, there is also a large variation in D6SR between individual trees, and across the time series. This may be expected as the crown of each tree has a unique position in the forest canopy, with a unique (and unknown) history. This influences canopy light exposure, leaf temperature, and stomatal conductance. The effect of diameter on D6SR indeed suggests that canopy exposure is an important driver of photosynthetic efficiency. Natural forest dynamics (e.g. branch fall, liana infestation, and canopy openings) can also influence canopy exposure, and thus the D6SR ratios. To reduce the effect of differences between trees, we chose to analyse the time series of D6SR for individual trees and include individual trees as a random variable. Yet, D6SR values within trees remain highly variable. Thus, measurements of D6SR of natural forest trees appear to be inherently more variable than those in agricultural or experimental systems (Ehlers *et al*., 2015), likely because the latter are shorter‐lived and grow under more uniform conditions. Increased sample sizes in future D6SR studies on trees are therefore necessary to confirm our findings. As D6SR is a relatively new method (Ehlers *et al*., 2015; Serk *et al*., 2021a,b), downstream fractionation of D6SR from leaf to wood, as seen for ^13^C isotopes (Gessler & Ferrio, 2022), has not yet been studied. We expect downstream fractionation might be limited as both D6^S^ and D6^R^ sugar molecules have the same molecular weight. For this study, we assume that if any fractionation is present, this is constant over time and therefore did not influence our estimates of *C*_a_ and diameter effects.

### Interpreting *C*_a_ effects on D6SR isotopomer ratios

The effect of *C*_a_ rise on plant productivity is generally expected to be positive. In line with Hypothesis 1, we found that D6SR is negatively associated with *C*_a_, corresponding to an increase in photosynthetic efficiency. Increases in forest canopy photosynthesis with *C*_a_ also follow from other lines of empirical evidence. First, FACE experiments on mature trees in temperate zones consistently show an increase in light‐saturated photosynthesis at the leaf level in response to increased *C*_a_ (Ainsworth & Long, 2005; Ellsworth *et al*., 2017; Jiang *et al*., 2020), without any apparent changes in canopy transpiration (Gimeno *et al*., 2018). However, all FACE experiments implement a stepwise increase in *C*_a_, which does not reflect realistic scenarios (Luo, 2001; Hendrey & Miglietta, 2006), and FACE experiments have yet to start in tropical forests (AmazonFace; Lapola & Norby, 2014). Second, tree‐ring‐derived ^13^C isotope studies show consistent increases in iWUE in tropical trees (Hietz *et al*., 2005; Brienen *et al*., 2011; Nock *et al*., 2011; van der Sleen *et al*., 2015; Rahman *et al*., 2020), which can be due to an increase in photosynthesis, a reduction in water use, or both. To evaluate the physiological effects of *C*_a_, the proxy of photosynthetic efficiency based on D6SR has an advantage over that of iWUE based on ^13^C isotopes, as it directly reflects photosynthetic processes and enzymes involved in the photorespiratory and photosynthetic pathway (Rinne‐Garmston *et al*., 2022) and is not influenced by the complex dynamics of stomatal conductance.

As D6SR is a new proxy, we place our results in a broader perspective by calculating β values of responses to *C*_a_ of D6SR, *V*_o_/*V*_c_, *C*_*i*_, and iWUE following Walker *et al*. (2020). These β estimates provide scaled values for the effect size of *C*_a_ rise, allowing comparison between studies using similar indices but different study designs. A β estimate of 1 (or −1) indicates a direct proportionality of the *C*_a_ response; values greater than one suggest a stronger response, while values less than one indicate a weaker response. For the *C*_a_ effect on D6SR, we find β = −0.094 (for median‐sized trees), which is slightly lower than β values for annual plants (Ehlers *et al*., 2015). The decreased D6SR ratio with rising *C*_a_ can be caused by suppression of photorespiration (oxygenation), increasing photosynthesis (carboxylation), or a combination of both. Based on photosynthetic modelling with fixed photosynthetic parameters (Fig. S14; Farquhar *et al*., 1980; Farquhar & Sharkey, 1982; Farquhar & Von Caemmerer, 1982), a 36% increase in *C*_a_ (this study) and *C*_i_, leads to a 26% decrease in *V*_o_/*V*_c_ (β = −0.98), coupled with a 32% increase in gross photosynthesis (β = +0.90). This theoretical decrease in *V*_o_/*V*_c_ is mostly driven by a strong increase in carboxylation and a smaller concomitant decrease in oxygenation (Fig. S1). Empirically, D6SR correlates strongly with *V*_o_/*V*_c_ for sunflowers in an experimental setting (Ehlers *et al*., 2015). If we assume this same empirical relationship for our study, our D6SR results translate into an 18% decrease in *V*_o_/*V*_c_ (31% lower than expected, β = −0.64), which would lead to a 22% increase in gross photosynthesis (same 31% lower, β = +0.64). These β values are similar to those for GPP from other studies (Walker *et al*., 2020). Finally, ^13^C isotope measurements for one of our study sites (Thailand; van der Sleen *et al*., 2015) show a 44% increase in *C*_i_ (β = +1.20) and a 23% increase in iWUE (β = +0.68) for *T. ciliata* trees of 27 cm DBH in response to the same 36% *C*_a_ rise (Fig. S15). Taken together, the similarity of observed and theoretical increases in *C*_i_, photosynthetic efficiency, and iWUE provide empirical evidence that *C*_a_ rise has likely led to increased canopy photosynthesis for our study species during the past century, although underlying assumptions make the magnitude of this estimate less certain.

### Effect of tree size on D6SR isotopomer ratio

As trees grow from saplings into adults, they experience large changes in microclimate, including gradients in light (Monsi & Saeki, 2005; Brienen *et al*., 2022), temperature (Hinckley *et al*., 2011) and hydraulic demands (Koyama *et al*., 2021; Fernández‐de‐Uña *et al*., 2023), which may result in changes in the ratio of photosynthesis to photorespiration. Following Hypothesis 2, that canopy exposure reduces photosynthetic efficiency, we found that D6SR increases with tree diameter, analogous to a decreased photosynthetic efficiency. This positive association is less strongly supported under a linear assumption (PP 94%) than under a log‐linear assumption (PP 96%), suggesting that a linear model may not accurately describe the relationship between D6SR and tree size. Assuming a saturating relationship, D6SR approaches an asymptote at *c*. 60 cm in diameter, coinciding with the moment trees of our study species reach the maximum height (Fig. S11A) and usually hold dominant canopy positions (Fig. S11B). These results are consistent with ^13^C studies, which often find a decrease in *C*_*i*_ with tree height at the leaf and stem level (McDowell *et al*., 2011) and also support the asymptotic change, as is often found for the association between iWUE and tree diameter (Brienen *et al*., 2017), although a strong ontogenetic effect is not always observed for temperate light‐sensitive species (McCarroll *et al*., 2020). ^13^C measurements for *T. ciliata* in our Thailand site show that *C*_*i*_ is consistently lower in canopy trees than in subcanopy trees, and this offset remains across a range of *C*_a_ (Fig. S15B) (van der Sleen *et al*., 2015). Decreasing *C*_*i*_ is expected to decrease photosynthetic efficiency (Farquhar *et al*., 1980) (Fig. S1A), coinciding with the observed increase in D6SR. The decrease in leaf internal *C*_*i*_ based on ^13^C, as well as the decreased photosynthetic efficiency found in this study, could be explained by stomatal closure (due to hydraulic limitations; McDowell *et al*., 2011) and by increased rates of photosynthesis causing reduction in *C*_*i*_ (photosynthetic drawdown; Francey & Farquhar, 1982; Lloyd *et al*., 2009). Canopy exposure plays a key role as photosynthetic efficiency decreases with diameter; however, this does not imply that net photosynthesis (Rijkers *et al*., 2000; Kenzo *et al*., 2006; Sterck & Schieving, 2011; Lamour *et al*., 2023) and growth (Sillett *et al*., 2010; Stephenson *et al*., 2014) also decrease with tree size.

The associations of D6SR with diameter and *C*_a_ were opposing, complicating the interpretation of *C*_a_ effects for trees, as all extant large trees will have grown from juvenile to adult across a gradient of *C*_a_. Using simulations from our gMM model, we found that a ‘hypothetical’ tree with a constant diameter increment, growing under exponential *C*_a_ rise (data from past century), will first experience a decrease in photosynthetic efficiency, resulting in a maximum D6SR (at *c*. 60 cm), after which *C*_a_ starts increasing photosynthetic efficiency (Fig. 4). With increasing tree diameter (and thus crown exposure), photosynthesis likely shifts from light‐limited to carboxylation‐limited, due to high light exposure (Farquhar *et al*., 1980; Farquhar & Von Caemmerer, 1982; Lambers & Oliveira, 2019) (Fig. S1) and increasing hydraulic demands. Carbon limitation of photosynthesis in fully exposed crowns would explain the increase in photosynthetic efficiency with *C*_a_ rise, as light and hydraulic limitation should stabilize after trees reach dominant canopy positions. While this study was done for only one tree species, the consistent decrease in ^13^C‐based *C*_*i*_ with tree height for multiple (tropical) angiosperm species (McDowell *et al*., 2011) suggests that a similar decrease in photosynthetic efficiency with canopy exposure may be expected for other broadleaf tree species. The effect of diameter on *C*_*i*_ in stem wood reflects the signature of the upper canopy layer (Schleser, 1990; Brienen *et al*., 2017), suggesting that the upper canopy layer produces the majority of photosynthates used in stem wood formation (Lamour *et al*., 2023). This has important implications for the effects of future *C*_a_ rise, as, based on our results, photorespiration may be highest in the upper canopy layer. We therefore expect CO_2_ fertilization to remain important for large trees for a long time, but to be less important for smaller unexposed trees.

### Effects of climate on D6SR isotopomer ratio

Based on other studies of isotopomers (Serk *et al*., 2021b), photosynthetic theory (Lambers & Oliveira, 2019), and ^13^C isotopes (van der Sleen *et al*., 2022), we expected photosynthetic efficiency to be lower when temperature is high and precipitation is low (Hypothesis 3). A positive effect of temperature on D6SR, and thus a decrease in photosynthetic efficiency, is possible from our data, yet our results did not provide strong support for these effects (83% PP). Potential explanations for the lack of consistent climate associations include the relatively small number of sites (three), tree size differences between sites (which could have concealed climatic effects), and local variation in growing location between trees combined with limited numbers of replicated trees per site. The strength of observed *C*_a_ effects could be decreased by long‐term increases in *T*_day_; this is, however, unlikely given that a historical increase in *T*_day_ was only observed for the Bangladesh site (Fig. S16). Precipitation did show changes over time for the Australian and Bangladeshi sites (Fig. S16), though the effects of precipitation remained highly uncertain.

### Concluding remarks and future outlook

We conclude that the photosynthetic efficiency of *T. ciliata* trees increased in association with ongoing anthropogenic *C*_a_ rise while increasing tree diameter decreased photosynthetic efficiency. This study adds an independent line of empirical evidence for a *C*_a_‐induced increase in the photosynthetic efficiency of tropical forest canopies, in addition to *C*_i_ rise from tree‐ring ^13^C isotopic studies (van der Sleen *et al*., 2022), GPP rise from carbonyl sulphide (COS) measurements (Campbell *et al*., 2017; Cernusak *et al*., 2019), and GPP rise from remotely sensed solar‐induced Chl fluorescence (SIF; Song *et al*., 2022). These methods differ in temporal scales covered (decadal for SIF, centennial for the others), spatial resolution (global for COS, spatially explicit for the others), and attribution to photosynthesis rise (indirect for *C*_*i*_, direct for the others). To our knowledge, isotopomers are currently the only method available to estimate changes in photosynthetic efficiency at centennial scale, for specific forests. However, the spatial coverage of D6SR is very limited at present and a broader application is strictly dependent on the ability to date samples (using tree rings or radiocarbon dating).

Our results open up the following new avenues to study photosynthetic responses to long‐term *C*_a_ rise in trees. (1) Disentangling the effects of crown exposure and *C*_a_ requires more scrutiny, for instance, by measuring D6SR in trees of different sizes in a FACE experiment, or by measuring tree‐ring‐based D6SR in fixed diameter classes from small to large DBH, that grew over a gradient of *C*_a_ (following van der Sleen *et al*., 2015). (2) The effects of canopy exposure on D6SR require further study. While it is likely that increasing irradiance (due to canopy exposure) is a strong driver, the role of hydraulic limitation should not be overlooked. Studies including trees with fully light‐exposed crowns, contrasted with trees of similar size growing in closed‐canopy forests, could help differentiate the effects of light and hydraulic limitation. (3) The implications of D6SR changes for the ratio of oxygenation and carboxylation (*V*_o_/*V*_c_) can be further explored by measuring leaf *V*_o_/*V*_c_ and leaf D6SR on different species grown at different temperatures in glasshouses as well as in canopy trees. The new AmazonFACE experiment offers opportunities to do so *in situ* (Lapola & Norby, 2014). (4) The effects of climate could be further explored, both by glasshouse studies on tropical tree saplings grown under different temperature and water regimes and on canopy trees by including larger temperature and precipitation gradients. (5) Lastly, the generality of our findings for other (tropical) tree species needs to be established. The strength of the *C*_a_ effect and the shape of the diameter curve may differ, due to differences in shade tolerance, deciduousness, and strategies of stomatal control. Thus, future studies on D6SR would benefit from including a range of different tree species to study how *C*_a_ rise has and will influence tree photosynthesis and productivity of tropical forests.

## Competing interests

None declared.

## Author contributions

SAZ, PAZ FJS, LH and JS designed the study. SAZ selected the included samples, and LH carried out the laboratory work and measurements. SAZ designed and implemented the statistical analyses with help from JAM‐V and AM. MR, MV and IH supplied the wood samples. SAZ, PAZ, FJS, LAC, AM and NPRA interpreted the results. SAZ drafted the first version of the manuscript, with the help of PAZ and FJS. All authors contributed to subsequent versions.

## Disclaimer

The New Phytologist Foundation remains neutral with regard to jurisdictional claims in maps and in any institutional affiliations.

## Supporting information


**Fig. S1** Farquhar simulations of *V*_o_, *V*_c_ and photosynthesis.
**Fig. S2** Distribution of DBH against *C*_a_ for included samples.
**Fig. S3** Reaction scheme illustrating the synthesis of glucose derivatives from cellulose.
**Fig. S4** Deuterium NMR spectrum.
**Fig. S5** Visualization of potential parameter combinations based on priors.
**Fig. S6** Density plots of the posterior distribution and trace plots for gMM model.
**Fig. S7** Posterior probability against posterior draws.
**Fig. S8** Predictions for site specific models.
**Fig. S9** Predictions for the gMM model based on sample means.
**Fig. S10** *C*_a_ effects on the 1/*C*_a_ axis.
**Fig. S11** Tree architecture of *Toona ciliata*.
**Fig. S12** Graphical comparison between gMM model and alternative models.
**Fig. S13** Joint effects of *C*_a_ and DBH based on linear and log‐linear model.
**Fig. S14** Farquhar simulation of photosynthesis.
**Fig. S15** *C*_i_ and iWUE estimates based on ^13^C isotopes.
**Fig. S16** Trends in climate data.
**Methods S1** Glucose derivative preparation for deuterium NMR.
**Methods S2** Statistical model description.
**Table S1** Model comparison.
**Table S2** Estimates for the generalized mixed Michaelis–Menten model.
**Table S3** Estimates for alternative models.
**Table S4** Estimates for site specific models.
Please note: Wiley is not responsible for the content or functionality of any Supporting Information supplied by the authors. Any queries (other than missing material) should be directed to the *New Phytologist* Central Office.

## Data Availability

Data used in this study are available on Zenodo (doi: 10.5281/zenodo.14280864).
