# Supplementary material for: Centennial‐scale atmospheric CO2 rise increased photosynthetic efficiency in a tropical tree species
Source: New Phytol. 2025 Feb 12;246(1):131–43. doi: 10.1111/nph.20358 (PMC11883053; doi:10.1111/nph.20358)
Supplement: Supplementary file 1 — Fig. S1 Farquhar simulations of V o, V c and photosynthesis. Fig. S2 Distribution of DBH against C a for included samples. Fig. S3 Reaction scheme illustrating the synthesis of glucose derivatives from cellulose. Fig. S4 Deuterium NMR spectrum. Fig. S5 Visualization of potential parameter combinations based on priors. Fig. S6 Density plots of the posterior distribution and trace plots for gMM model. Fig. S7 Posterior probability against posterior draws. Fig. S8 Predictions for site specific models. Fig. S9 Predictions for the gMM model based on sample means. Fig. S10 C a effects on the 1/C a axis. Fig. S11 Tree architecture of Toona ciliata. Fig. S12 Graphical comparison between gMM model and alternative models. Fig. S13 Joint effects of C a and DBH based on linear and log‐linear model. Fig. S14 Farquhar simulation of photosynthesis. Fig. S15 C i and iWUE estimates based on 13C isotopes. Fig. S16 Trends in climate data. Methods S1 Glucose derivative preparation for deuterium NMR. Methods S2 Statistical model description. Table S1 Model comparison. Table S2 Estimates for the generalized mixed Michaelis–Menten model. Table S3 Estimates for alternative models. Table S4 Estimates for site specific models. Please note: Wiley is not responsible for the content or functionality of any Supporting Information supplied by the authors. Any queries (other than missing material) should be directed to the New Phytologist Central Office. [file NPH-246-131-s001.pdf]

New Phytologist Supporting Information

Article title: Centennial-scale atmospheric CO_2 rise increased photosynthetic efficiency in a tropical tree species

Authors: Sophie A. Zwartsenberg[1], Frank J. Sterck[1], Lenny Haddad[2], Jürgen Schleucher[2], Niels P.R. Anten[3], Alejandro Morales[3], Lucas A. Cernusak[4], José A. Medina-Vega[5], Mizanur Rahman[6, 7], Mart Vlam[1,8], Ingo Heinrich[9], Pieter A. Zuidema[1]

Corresponding author: Sophie A. Zwartsenberg (sophie.zwartsenberg@gmail.com)

Article acceptance date: 30 November 2024

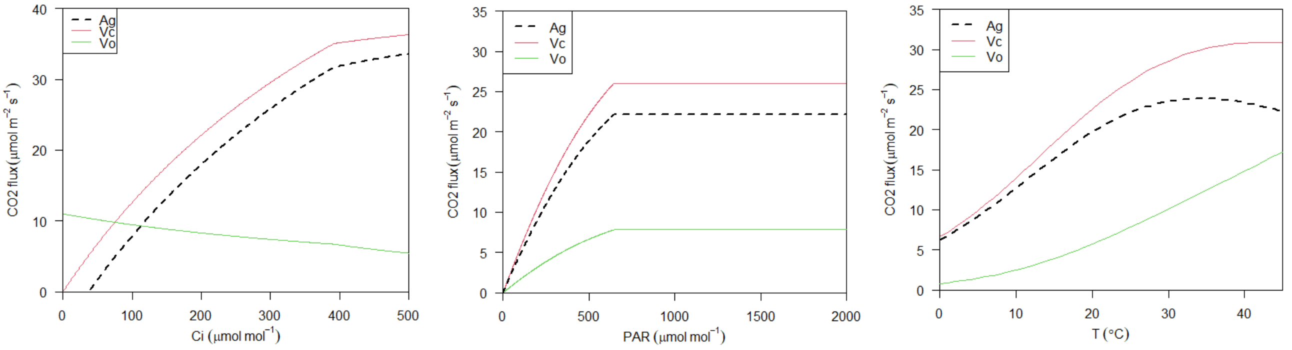

Figure S1. Simulated relationship between the rates of oxygenation (Vo), carboxylation (Vc), and gross photosynthesis (Ag) against intercellular CO_2 (C_i), incident photosynthetically active radiation (PAR), and leaf temperature (T). Simulations are based on the model by Farquhar *et al.* (1980).

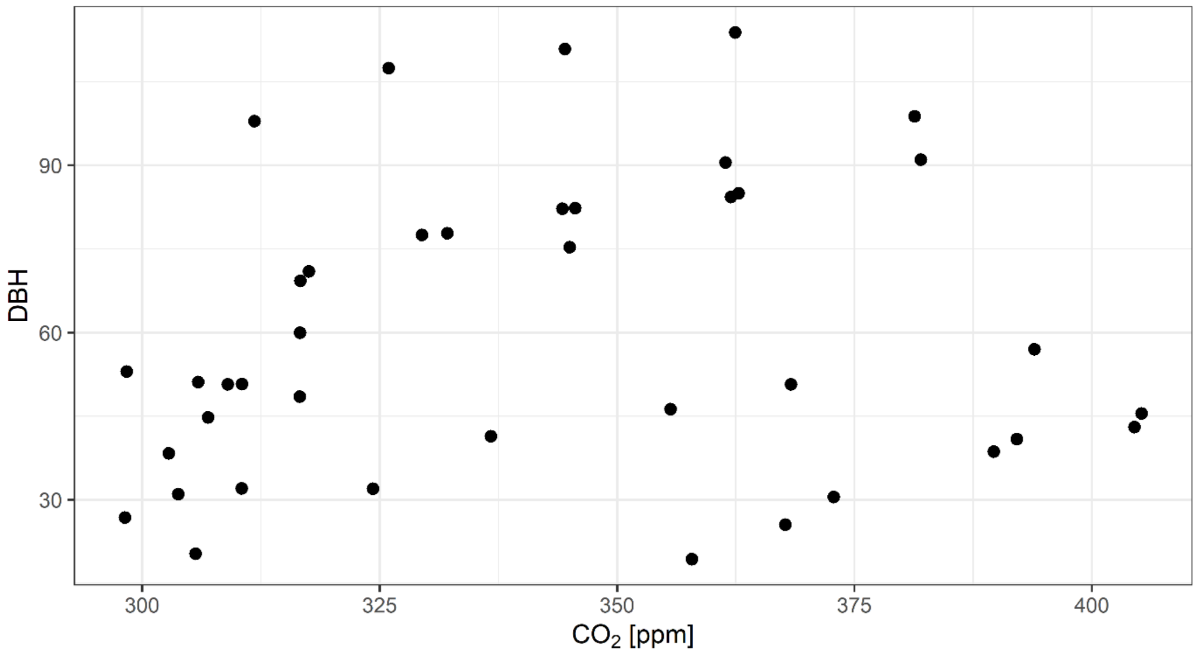

Figure S2. Distribution of mean Diameter Breast Height (DBH) and mean CO_2 of all included samples in this study.

Methods S1 - Glucose derivative preparation for deuterium NMR

For glucose derivatization, we followed the protocol of Schleucher *et al.* (1999) and Betson *et al.* (2006) for samples containing 250 mg of glucose. Glucose was transformed into 1,2-5,6-di-O-isopropylidene-α-D-glucofuranose (derivative 1) by reacting it with 30 mL of acetone and 1.26 mL of 96% H_2SO_4 for 4 hours. The acid was added in three portions: 180 µL at the beginning, 540 µL after 1 hour, and 540 µL after 3 hours. The solution was vacuum-filtered into a flask containing a mixture of 75 g of ice and 45 mL of water. This solution was left at 0 °C for 16 hours (overnight) to selectively hydrolyze derivative 1 to 1,2-O-isopropylidene-α-D-glucofuranose (derivative 2). The hydrolysis was stopped by neutralizing the solution with $CaCO_3$. The solution was then filtered and evaporated, and the residue was dried under a vacuum.

To remove mannose monoacetonide, derivative 2 was dissolved in a 20 mL $NaH_2PO_4.2H_2O$ solution, and $NaClO_2$ was added as a solid in six steps (e.g., at 9 am and 9 pm) over 3 days, with the pH adjusted to 3.5. In the final step, the pH was adjusted to around 5-6 with $CaCO_3$, and the solution was quenched with Na_2SO_3 (equivalent to the sum of the added $NaClO_2$). The solution was neutralized with $CaCO_3$ to reach a pH of 7, filtered, and the water was evaporated. Derivative 2 was washed with 20 mL of ethanol, vacuum-filtered, and dried overnight.

Derivative 2 was converted to 3,6-anhydro-1,2-O-isopropylidene-α-D-glucofuranose (Derivative 3) by heating the mixture to 60°C in a water bath for 1.5 hours with four molar equivalents of 0.5 mol l-1 sodium methanoate in methanol and two molar equivalents of dimethyl carbonate. Derivative 3 was purified by column chromatography using silica gel and diethyl ether as a solvent. Fractions were checked using TLC, and the compound's purity was evaluated using ^1H NMR.

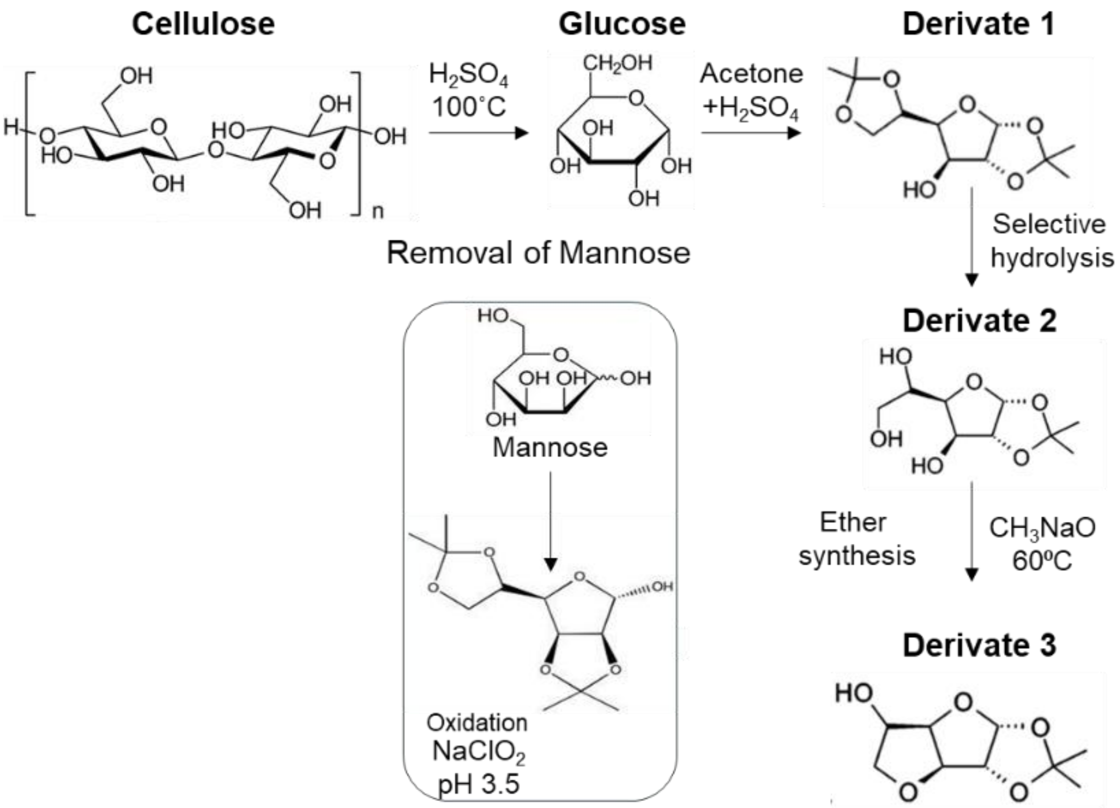

42 **Figure S3.** Reaction scheme illustrating the synthesis of glucose derivatives from cellulose.

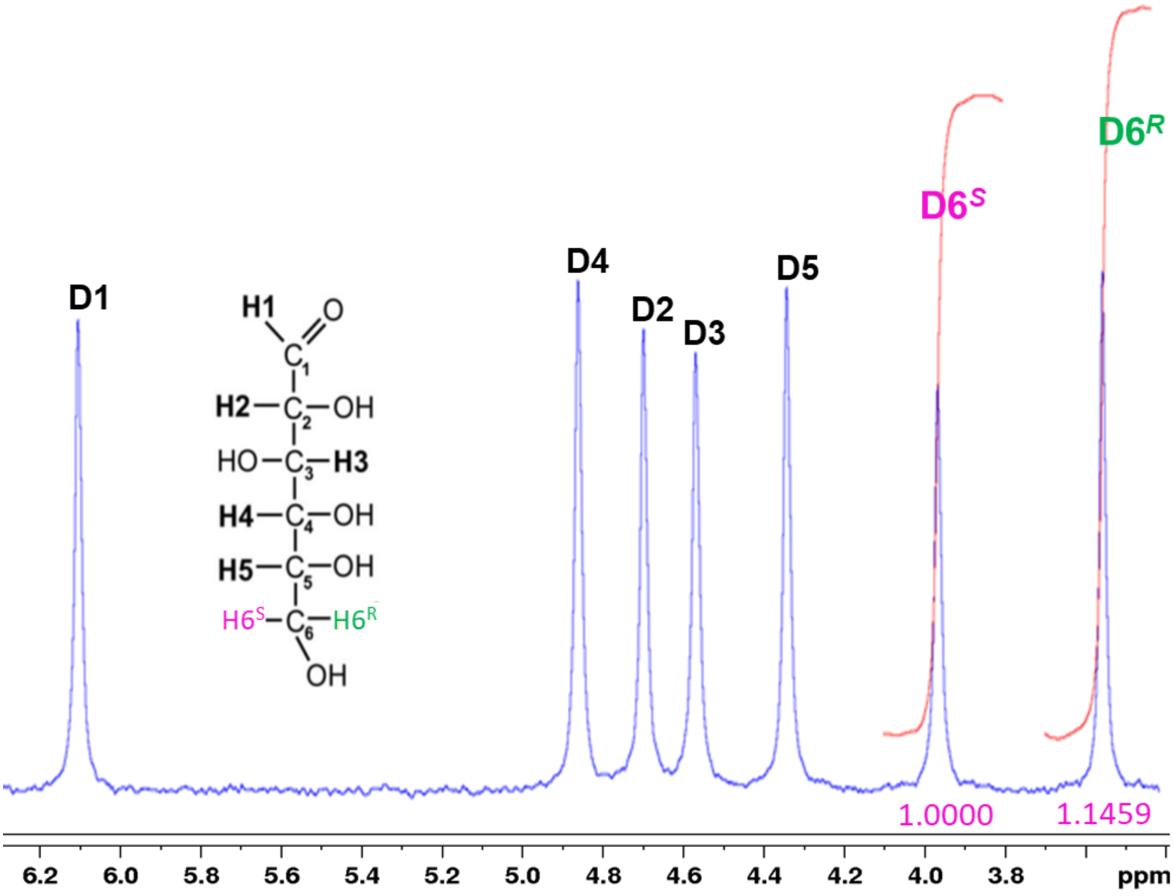

Figure S4. An example of a deuterium NMR spectrum of glucose derivative 3, with each signal representing one of the seven isotopomers of glucose (D1 – D6). The integrals of these signals are directly proportional to the abundances of the respective isotopomers.

Methods S2 Statistical model description

2.1 Main statistical model

To describe the nonlinear effects of diameter on the D6SR ratio we made use of a generalized Michaelis Menten function with the shape:

$$D6SR = \frac{a \cdot \text{diameter}}{b + \text{diameter}} + \sigma_{stc} \quad \text{(S1.1)}$$

Where D6SR is the ratio between glucose isotopomers D6S and D6R, a describes the asymptote that D6SR reaches with increasing tree diameter, *b* is the half-saturation parameter, and the residual standard deviation is given by σ_{stc}. The asymptote *a* is a function of:

$$a \sim \beta_{0a} + \beta_1 \text{CO}_2 + \beta_2 \text{T}_{\text{day}} + \beta_3 \text{precipitation} + S_{stc} + T_{tc} + C_c \quad \text{(S1.2)}$$

in which β_{0a} is the general intercept for the asymptote a, β_1 is the slope for the CO_2 effect, β_2 is the slope for the T_{day} effect and β_3 is the slope for the precipitation effect. Random intercepts for sample, tree, and country are given by S_{stc}, for sample nested within tree and country, T_{tc} for tree nested in country and C_c for country. Half saturation parameter *b* is described by:

$$b \sim \beta_{0b} + S_{stc} + T_{tc} + C_c \quad \text{(S1.3)}$$

where β_{0b} is the main intercept for *b* allowed to vary with random intercepts S_{stc}, for sample nested within tree and country, T_{tc} for tree nested in country and C_c for country.

2.2. Description of priors

For the intercept of variable a, a normal distribution with a mean (μ) of 1 and a standard deviation (σ) of 0.2 was assigned (*Normal*[1, 1.02]). The intercept term for variable *b* was assigned a gamma distribution with a shape of 2 and a scale of 2 (*Gamma*[2, 2]), these priors allow for a wide variety in shapes of the gMM model (Fig. S5). The β coefficients for the variables CO_2, T_{day}, and *precipitation* in *a* were assigned normal distributions with means of 0 and standard deviations of 0.5 (*Normal*[0,0.5]). Weakly informative priors were utilized for

the standard deviations of the random effects and the overall model error (σ), incorporating half-Cauchy distributions with a location parameter (μ) of 0 and a scale parameter (σ) of 1 (*half-Cauchy*[0, 1]).

2.3 Alternative models

We tested two different alternative models, namely a linear model and a model with a log transformation on diameter.

2.3.1 Linear model

$$D6SR = \beta_0 + \beta_1 \text{ diameter} + \beta_2 \, \text{CO}_2 + \beta_3 \, \text{T}_{\text{day}} + \beta_4 \text{ precipitation} + S_{stc} + T_{tc} + C_c + \sigma_{stc} \qquad \text{(S2)}$$

Where D6SR is a function of β_0 (the intercept) and $\beta_1, \beta_2, \beta_3,$ and β_4 the fixed effects on the standardized predictor variables for diameter, CO_2, T_{day}, and precipitation. With standard deviations for random intercepts S_{stc}, T_{tc}, C_c with the same nested design as equation S1, σ_{stc} represents the error term sigma.

2.3.2 Log-linear model

$$D6SR = \beta_0 + \beta_1 log_e(\text{diameter}) + \beta_2 \, \text{CO}_2 + \beta_3 \, \text{T}_{\text{day}} + \beta_4 \text{precipitation} + S_{stc} + T_{tc} + C_c + \sigma_{stc} \qquad \text{(S3)}$$

Same as for equation S2 except β_1 now acts on the log-transformed values of diameter.

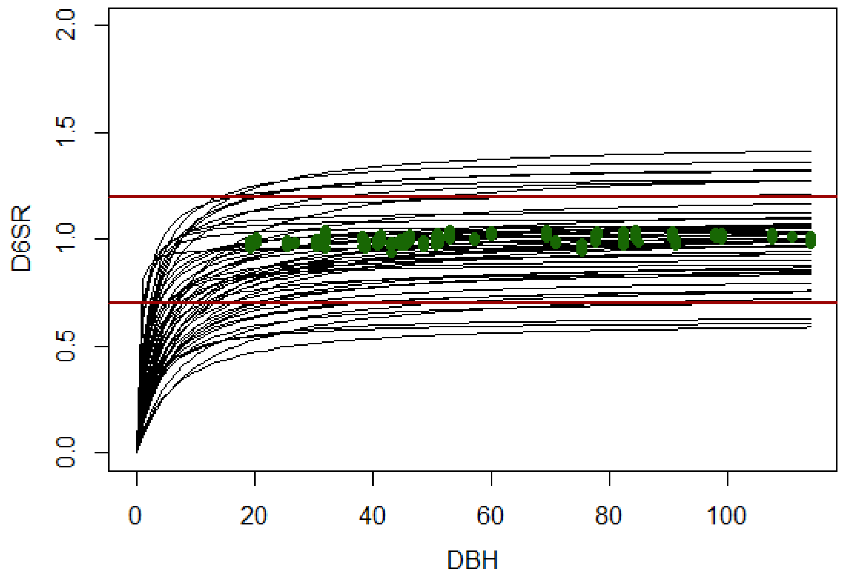

Figure S5. Random sampling of potential parameter combinations given the used priors for *a* and b of equation 1.1. Green points show the data of this study, red lines show the maximum and minimum D6SR values found in previous studies (Ehlers *et al.*, 2015).

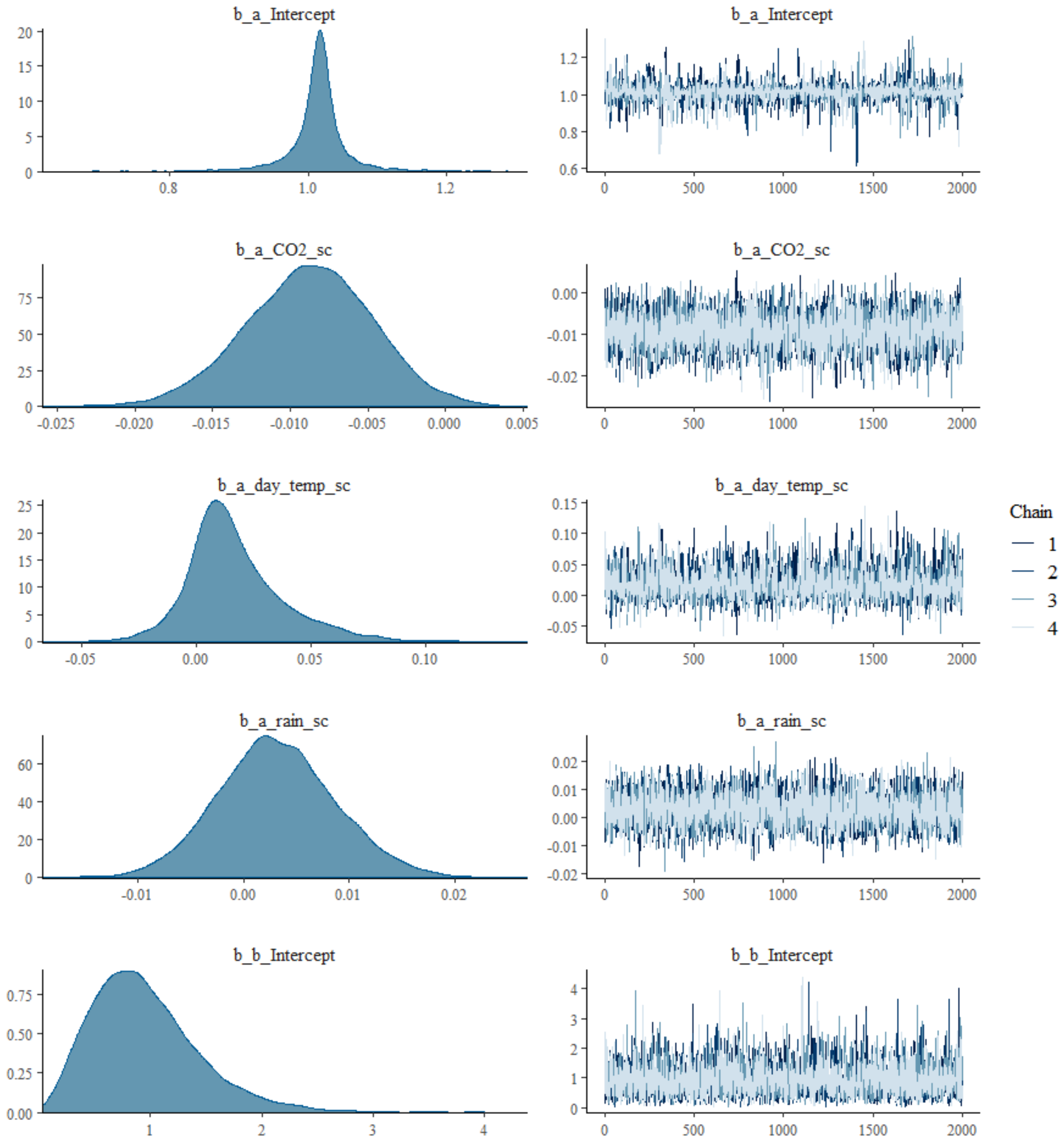

Figure S6. Density plots of the posterior distribution and trace plots for each model parameter for the gMM model (Eq. S1.1 – 1.3, Methods S2).

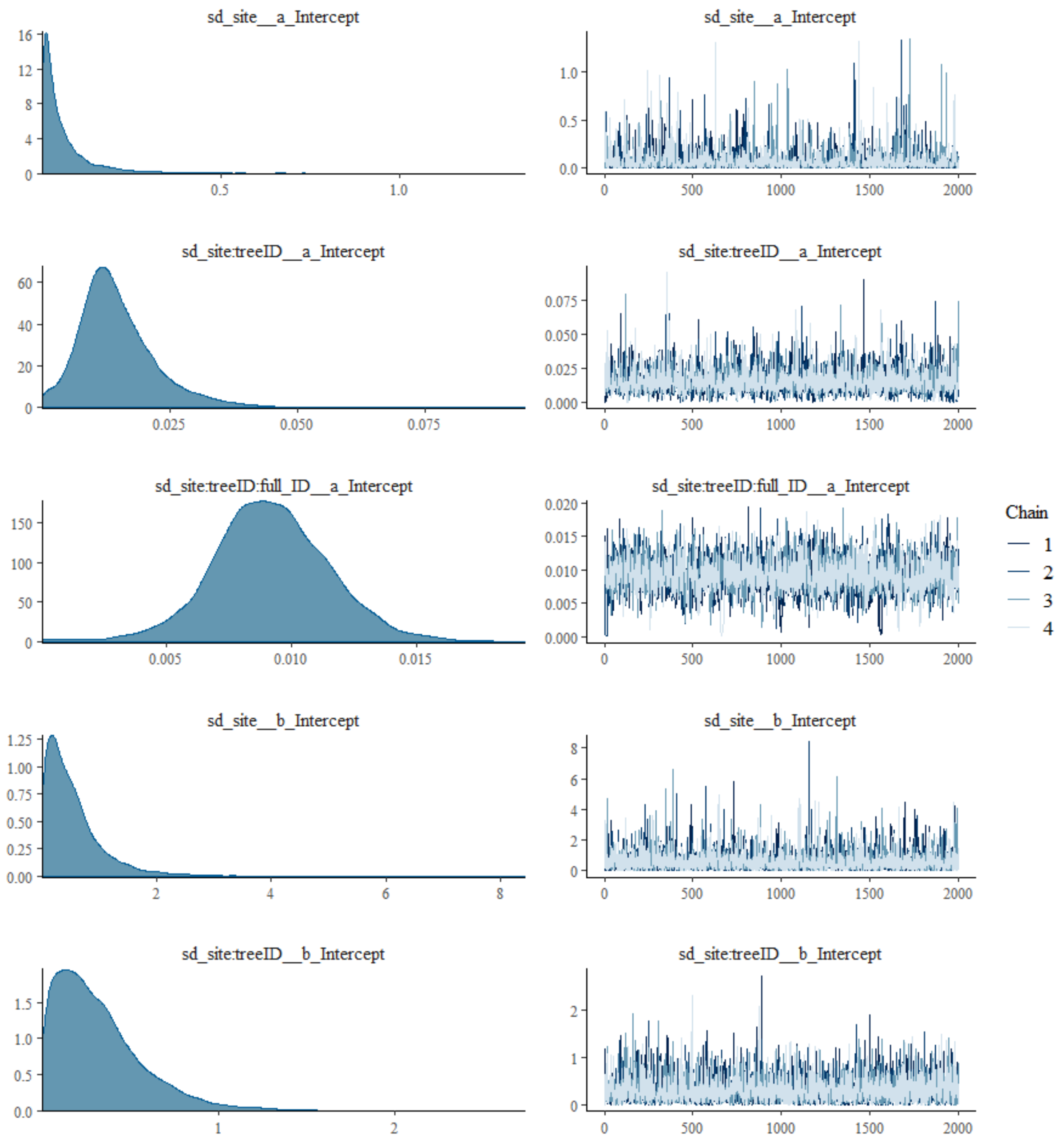

Figure S6. Continued

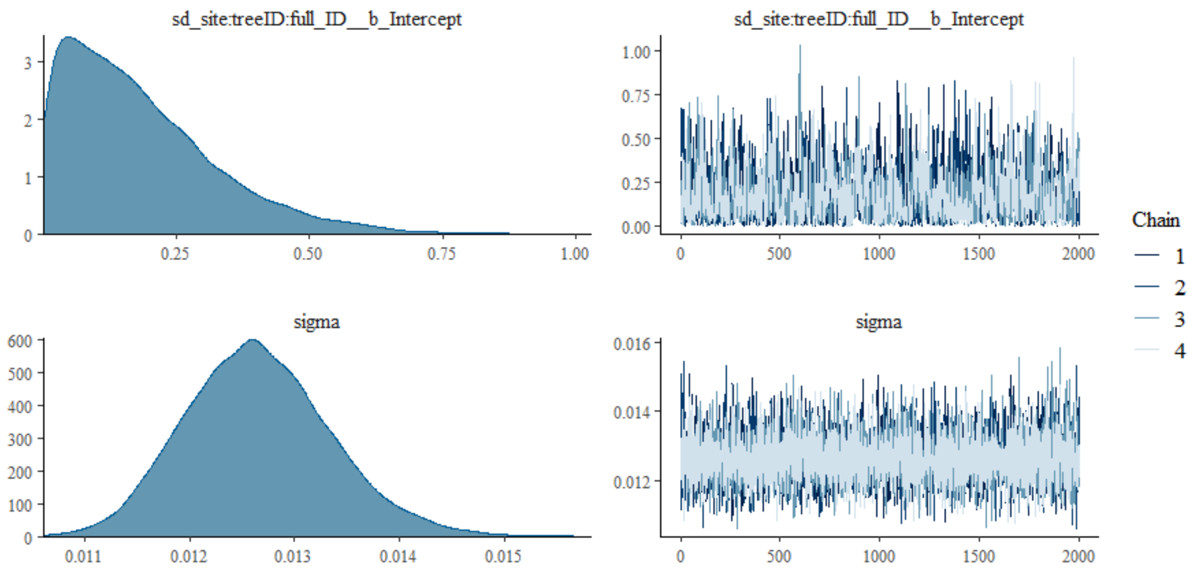

Figure S6. Continued

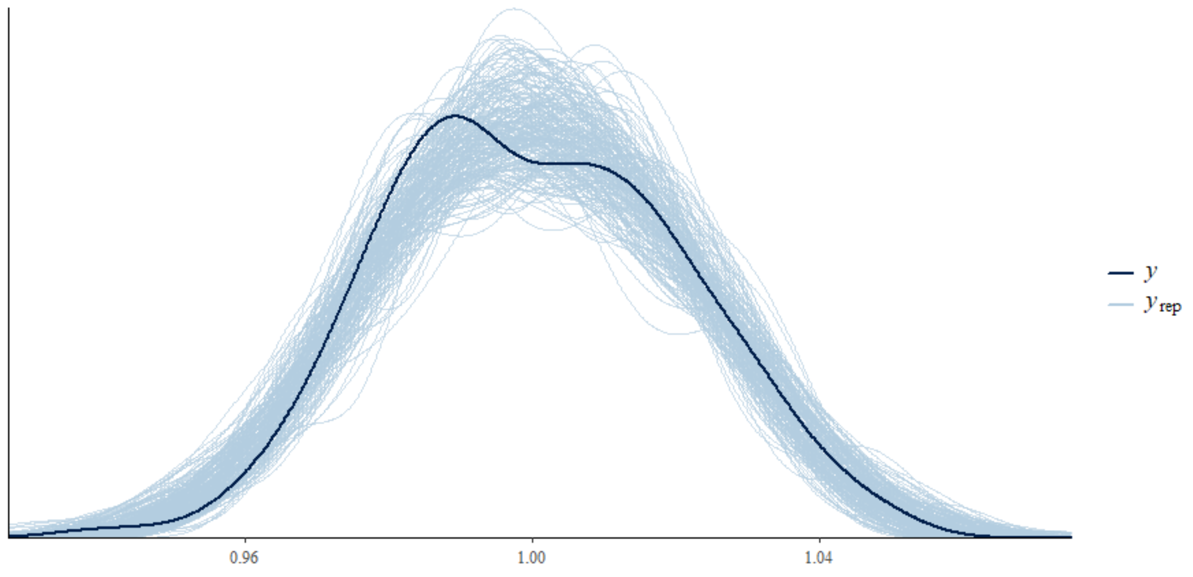

Figure S7. Posterior probability check, with y representing the Densitiy of observed D6SR and Yrep representing 200 posterior draws from the mixed gMM model. A good match between the two means the model is able to adequately capture the variation in the data.

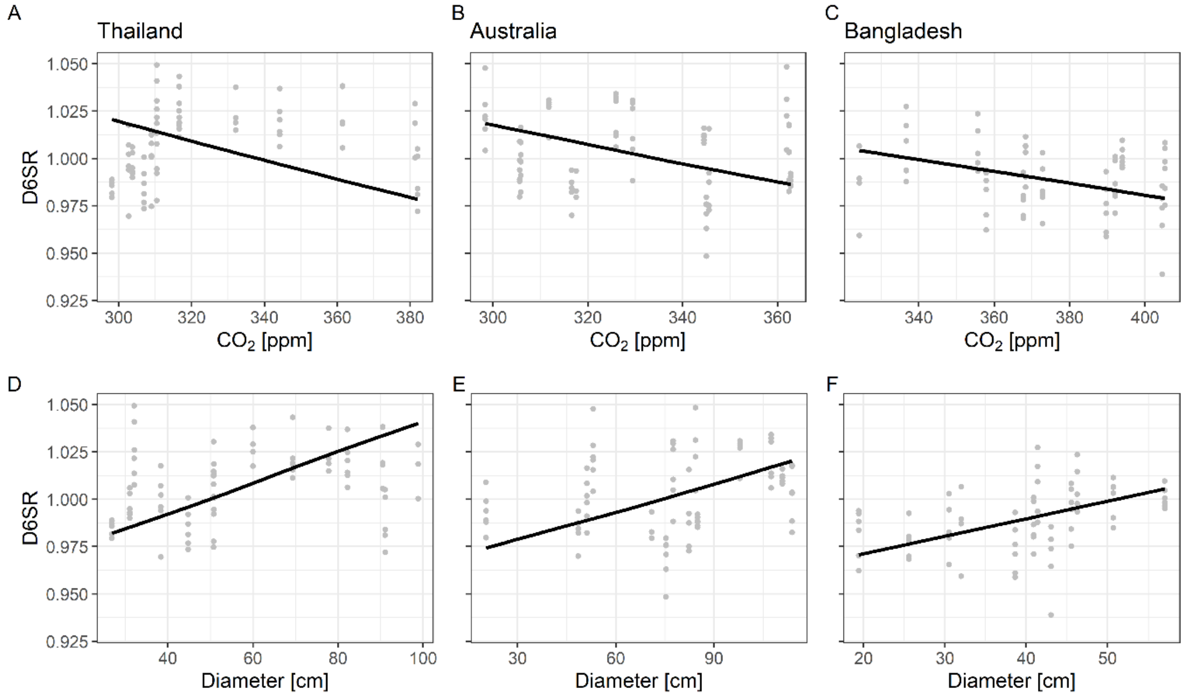

Figure S8. Model predictions for site specific models (presented in Table S4). Lines indicate the mean predicted effects of CO_2 and diameter on D6SR in *T.ciliata* trees for Thailand (A, D), Australia (B, E) and Bangladesh (C, F).

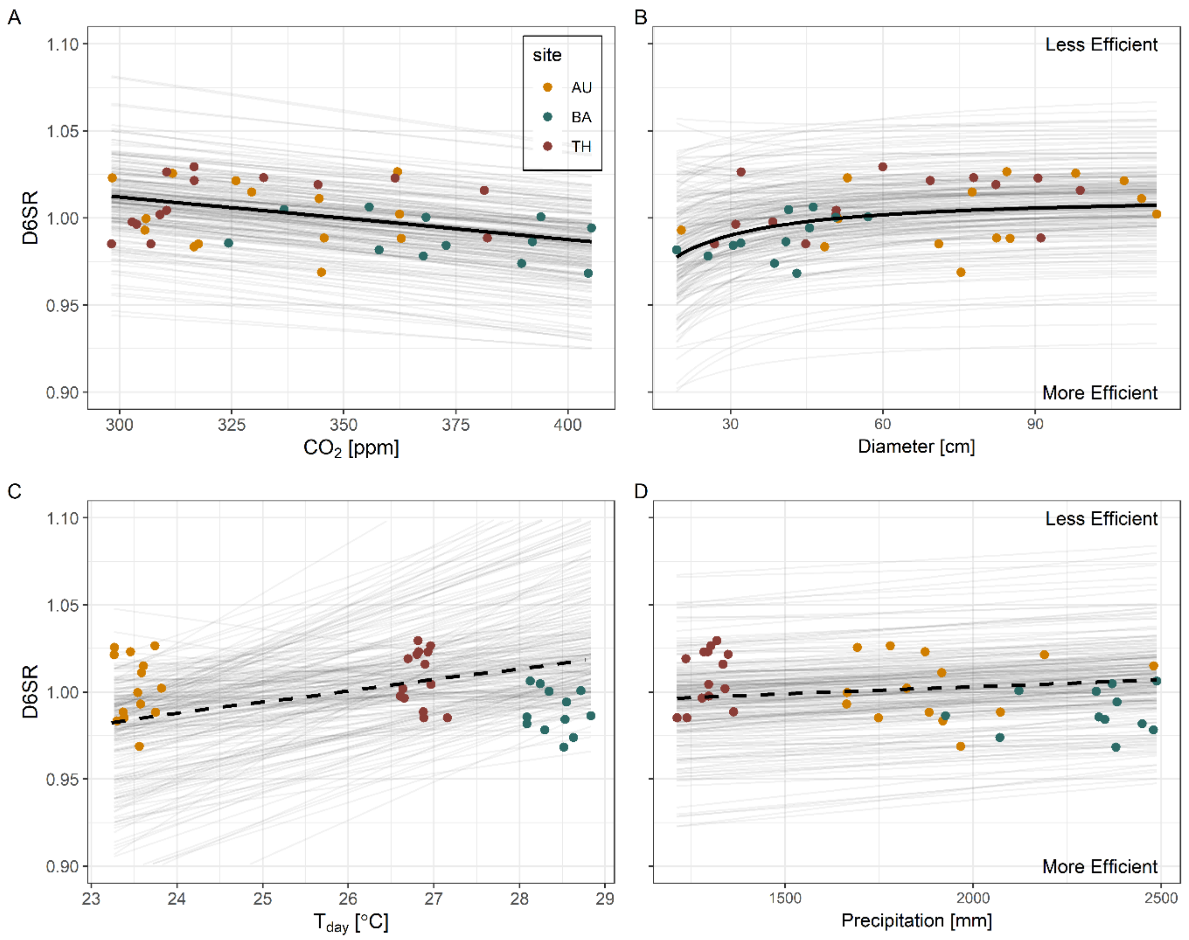

Figure S9. Predicted effects of atmospheric CO_2 (A), diameter (a measure of crown exposure, B), and climate (C, D) on the photorespiration-to-photosynthesis ratio (D6SR) in *T.ciliata* trees for three sampling sites (colours). Parameter estimates are based on the gMM model which was fitted on the sample means, this model thus includes a simpler random effect structure namely tree nested within site. Thick continuous lines indicate the predicted mean of the consistent associations (credible interval (CI) excludes zero and posterior probability (PP) > 95%), thick dashed lines are the predicted mean of effects that are not consistently associated (CI includes zero and posterior probability < 95%). Thin lines represent 200 draws from the posterior predictive distributions.

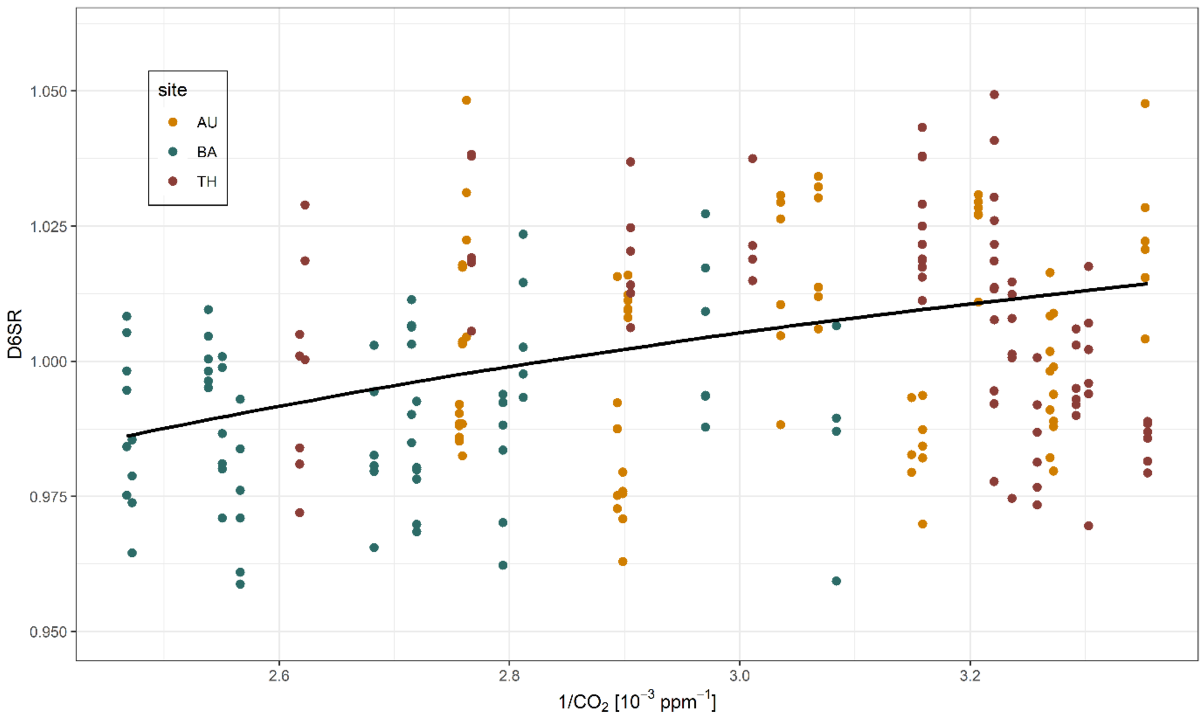

Figure S10. Predicted effects of atmospheric CO_2 on photorespiration-to-photosynthesis ratio (D6SR) for *T.ciliata* in three sampling sites (colours). Here shown on a 1/CO_2 axis, as done in the original publications on D6SR isotopomers. The slope of the line is 0.032, which is lower than the published slopes for crops (0.057 for sunflowers and 0.048 for sugar beet).

Table S1. Model comparison for models based on full data.

	**Michaelis Menten**	**Linear**	**Log-Linear**
Conditional R^2	0.641	0.637	0.637
Marginal R^2	0.327	0.337	0.314
LOOIC	-1278.7	-1279.2	-1279.9
WAIC	-1280.5	-1281.1	-1281.5

R^2 values were calculated using the r2_bayes function from the performance package (Daniel Lüdecke *et al.*, 2022), Watanabe-Akaike Information Criterion (WAIC) and Leave-One-Out Information Criterion (LOOIC) are based on the modelsummary package (Arel-Bundock, 2022).

Table S2. Estimates for the generalized mixed Michaelis-Menten model based on the equation presented in equations S1.1-1.3. Values represent the median of posterior parameter estimates and 95% credible intervals (in brackets) computed using quantiles. Percentages in italic after the square brackets indicate the posterior probability of the effect being positive or negative (following the sign of the estimate). Posterior probabilities are only provided for slope estimates, as comparison with zero is not meaningful for intercepts.

Parameter	Michaelis Menten (*full*)	Michaelis Menten (*means*)
a - Diameter	1.016 [0.908, 1.109]	1.012 [0.899, 1.101]
b - Diameter	0.890 [0.224, 2.144]	0.736 [0.204, 1.835]
a - CO_2	-0.009 [-0.017, -0.001] *99%*	-0.008 [-0.013, -0.003] *100%*
a - T_{day}	0.013 [-0.019, 0.069] *83%*	0.017 [-0.006, 0.052] *92%*
a - Precipitation	0.003 [-0.007, 0.014] *71%*	0.004 [-0.002, 0.011] *91%*
Sigma	0.013 [0.011, 0.014]	0.014 [0.013, 0.016]
a - Site	0.033 [0.001, 0.326]	0.034 [0.002, 0.351]
b - Site	0.412 [0.019, 2.044]	0.420 [0.023, 2.030]
a - Site: Tree	0.014 [0.003, 0.035]	0.017 [0.009, 0.040]
b - Site: Tree	0.266 [0.015, 0.950]	0.363 [0.025, 1.021]
a - Site: Tree: Sample	0.009 [0.004, 0.014]	
b - Site: Tree: Sample	0.152 [0.007, 0.543]	

The median of parameter estimates for the generalized Michaelis Menten model following Eq. S1 (supplementary methods 2). Coefficients for which the credible include zero are considered to be non-associated.

Table S3. Estimates for alternative models for D6SR (Eq. S2 – S4). The median of parameter estimates is presented with 95% credible intervals computed using quantiles. Percentages in italic after the square brackets indicate the posterior probability of the effect being positive or negative (following the sign of the estimate). Posterior probabilities are only provided for slope estimates, as comparison with zero is not meaningful for intercepts.

	**Linear (*full*)**	**Linear (*means*)**	**Log-Linear (*full*)**	**Log-Linear (*means*)**
Intercept	0.999 [0.767, 1.185]	0.998 [0.808, 1.160]	0.999 [0.835, 1.135]	0.999 [0.811, 1.146]
Diameter	0.008 [-0.002, 0.019] *94%*	0.006 [0.000, 0.012] *98%*		
Log Diameter			0.007 [-0.001, 0.016] *96%*	0.006 [0.001, 0.011] *99%*
CO_2	-0.009 [-0.020, 0.001] *96%*	-0.009 [-0.016, -0.002] *99%*	-0.009 [-0.018, 0.000] *97%*	-0.008 [-0.014, -0.003] *100%*
T_{day}	0.015 [-0.018, 0.072] *83%*	0.020 [-0.005, 0.057] *93%*	0.011 [-0.019, 0.067] *79%*	0.018 [-0.005, 0.054] *93%*
Precipitation	0.001 [-0.009, 0.012] *58%*	0.002 [-0.005, 0.008] *69%*	0.001 [-0.009, 0.012] *59%*	0.002 [-0.004, 0.008] *72%*
Sigma	0.013 [0.011, 0.014]	0.015 [0.014, 0.016]	0.013 [0.011, 0.014]	0.015 [0.013, 0.016]
Site	0.040 [0.001, 0.717]	0.042 [0.002, 0.552]	0.034 [0.001, 0.486]	0.041 [0.002, 0.589]
Site: TreeID	0.013 [0.004, 0.032]	0.015 [0.008, 0.034]	0.013 [0.004, 0.032]	0.015 [0.008, 0.036]
Site: Tree: Sample	0.010 [0.007, 0.015]		0.010 [0.007, 0.014]	

Values in square brackets represent the lower and upper 95% credible intervals.

Table S4. Parameter estimates for site specific models. To allow for inference with a lower number of observations, we removed the effects of T_{day} and precipitation. We also simplified the random effects which now only include re-measurement of samples. Effects were assumed to be linear for this analysis. The median of parameter estimates is presented with 95% credible intervals computed using quantiles. Percentages in italic after the square brackets indicate the posterior probability of the effect being positive or negative (following the sign of the estimate).

	**TH**	**AU**	**BA**
Intercept	1.000 [0.985, 1.016]	0.988 [0.971, 1.006]	1.017 [1.000, 1.033]
Diameter	0.021 [-0.004, 0.045] *95%*	0.013 [0.000, 0.026] *97%*	0.024 [0.009, 0.040] *99.6%*
CO_2	-0.016 [-0.042, 0.010] *89%*	-0.015 [-0.034, 0.004] *95%*	-0.010 [-0.018, -0.003] *99.6%*
Sigma	0.013 [0.011, 0.016]	0.012 [0.010, 0.015]	0.013 [0.011, 0.016]
Sample	0.015 [0.009, 0.026]	0.017 [0.011, 0.030]	0.006 [0.000, 0.015]

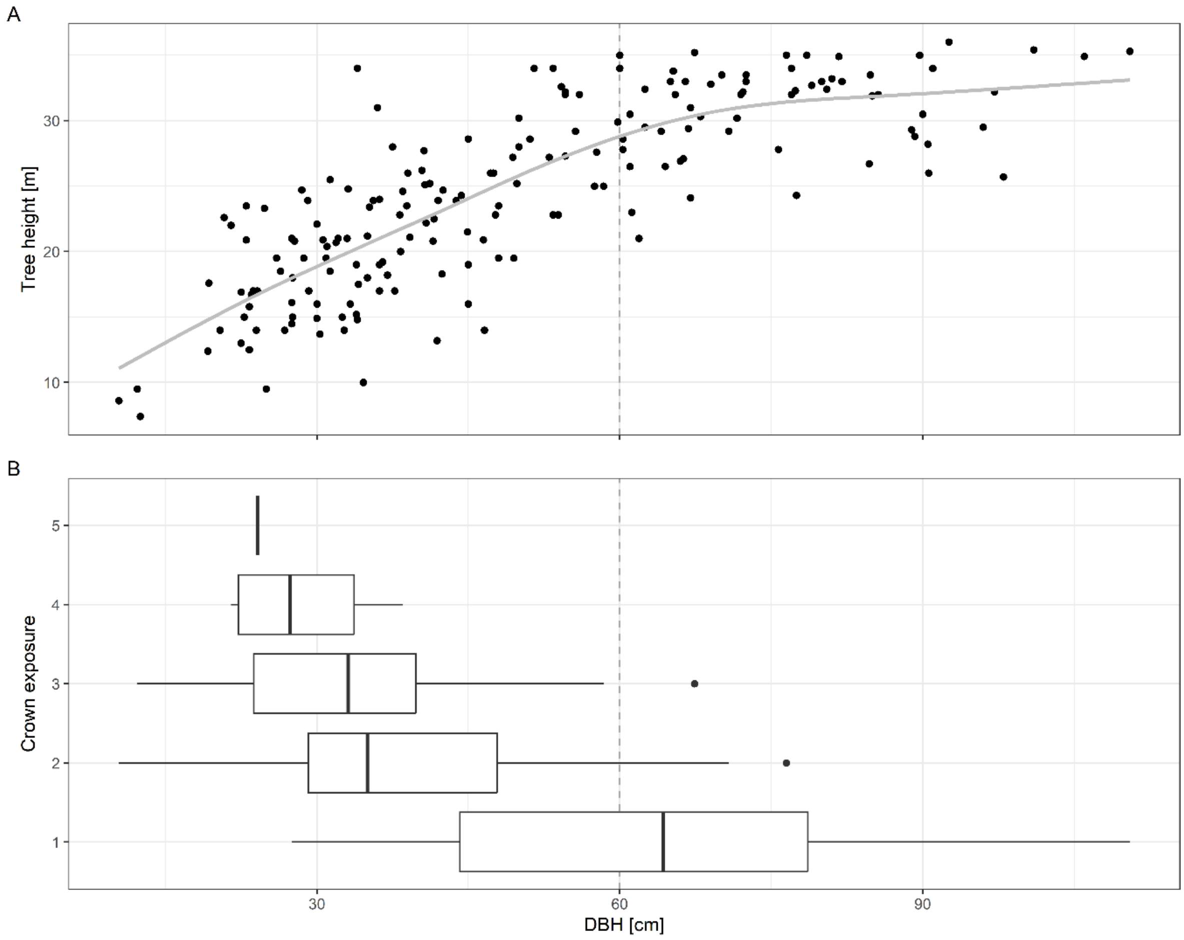

Figure S11. Tree architecture of *T. ciliata*. (A) Tree height versus diameter at breast height (DBH). (B) DBH against crown exposure, where crown exposure is measured in five classes with class “1” representing a fully exposed tree that gets light from the top and all four sides of the canopy, and “5” being fully shaded individuals that receive no direct light. Figures are based on field measurements from four different sampling locations in Northern Queensland, Australia, covering a range of climates. For boxplots, the central line represents the median, the edges of the boxes indicate the 25th and 75th percentiles, and the whiskers extend to the 5th and 95th percentiles. Outliers, if present, are shown as individual points.

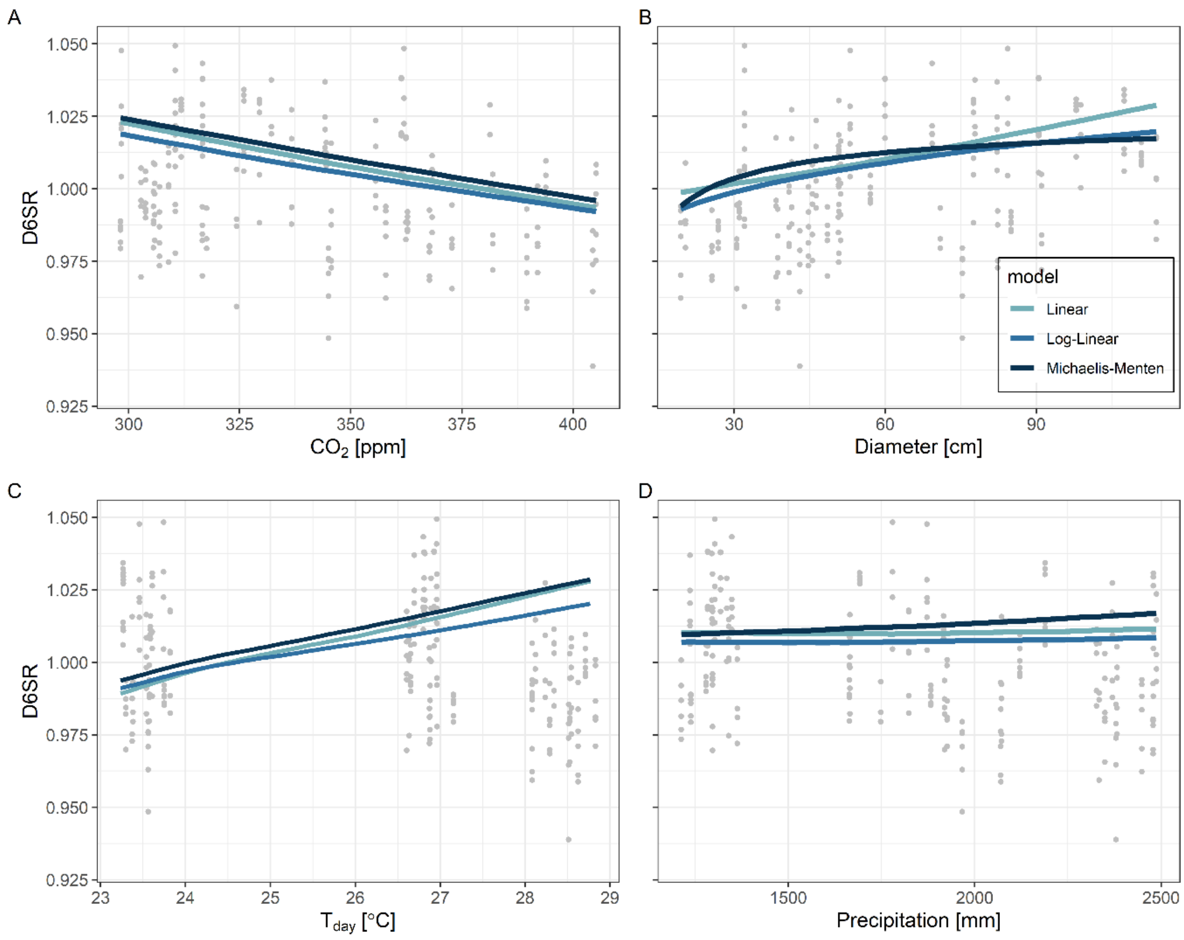

Figure S12. Comparison between the gMM model (Eq. S1.1 – 1.3) and the two alternative models (Eq. S2 & S3). Lines indicate the mean predicted effects of CO_2, diameter, T_{day} and precipitation for all three models (colours). Dots represent the raw data.

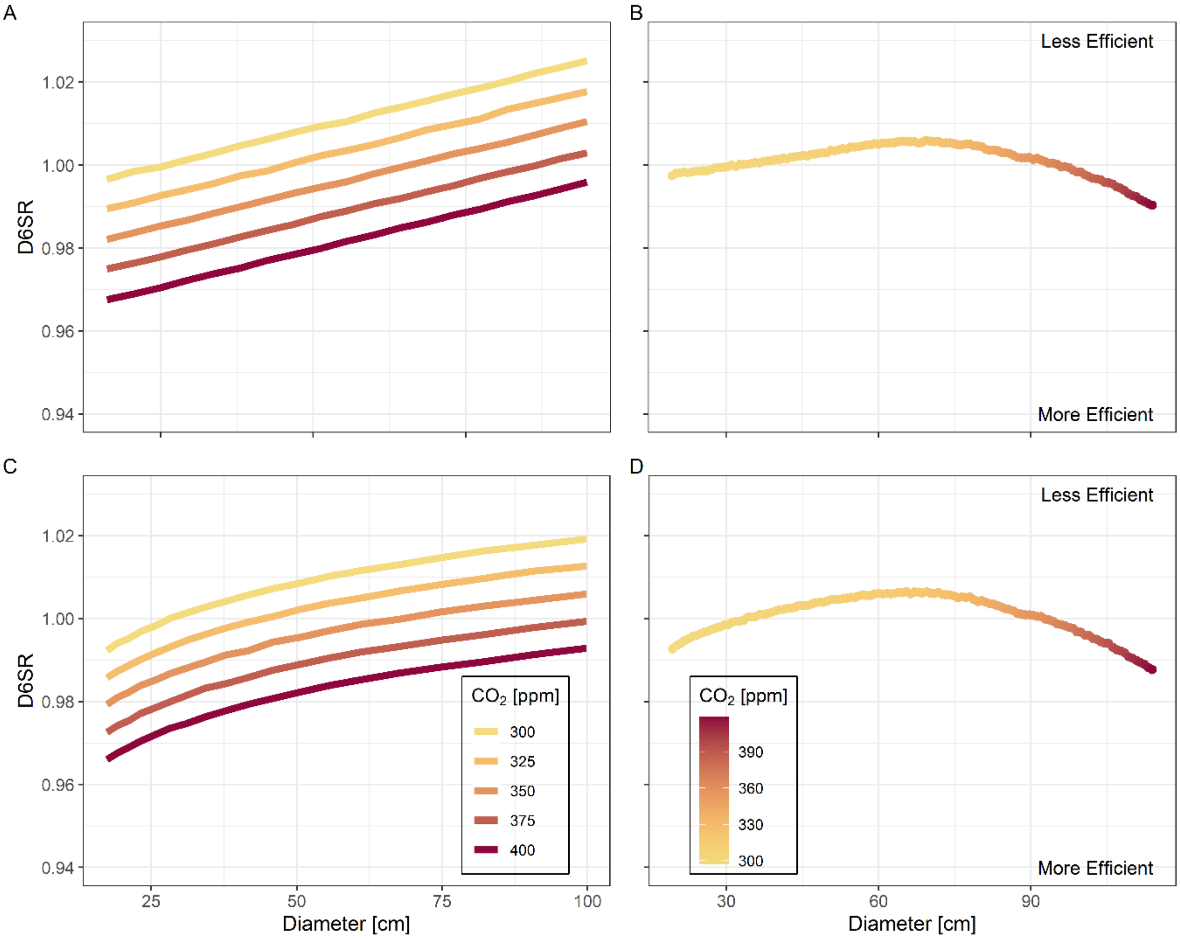

Figure S13. Predictions of joint effects of diameter and C_a on the photorespiration-to-photosynthesis ratio (D6SR) derived from the alternative linear model (A & B) and log-linear model (C & D) under (A & C) different constant C_a levels using a stepwise increase from 300 ppm (year) to 400 ppm (year), and (B & D) the effects of a simultaneous (constant) increase in diameter and 120 years of observed historic C_a increase (based on C_a data).

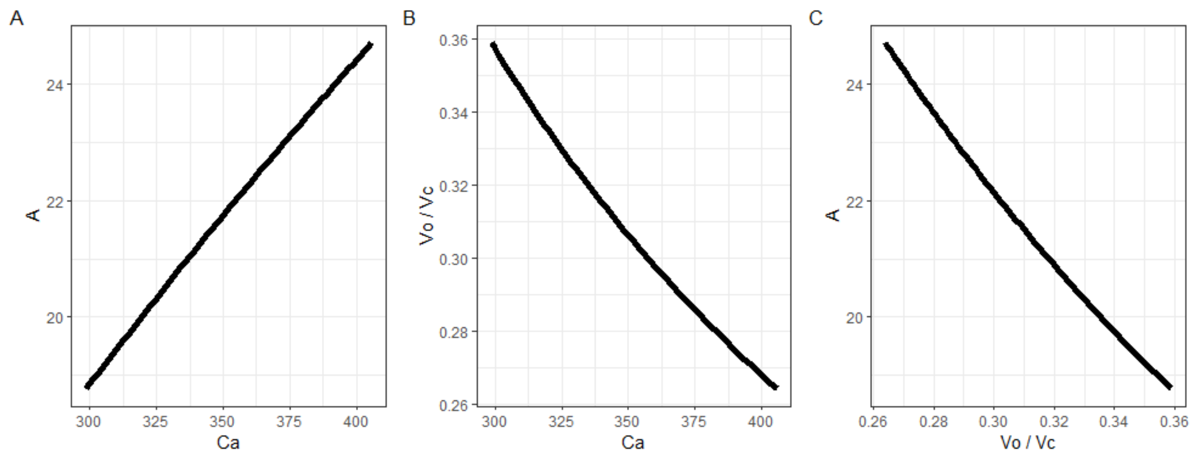

Figure S14. Simulated values of gross photosynthesis against C_a (A), Vo/Vc against C_a (B) and gross photosynthesis against Vo/Vc. Simulations are based on Farquhar *et al.* (1980) enzyme kinetics with parameters: Vcmax25 = 90, Ha_Vcmax = 65330, Jmax25 = 230, Ha_Jmax = 50000, Hd_Jmax = 2e6, S_Jmax = 650, KmC25 = 270, Ha_KmC = 80990, KmO25 = 165, Ha_KmO = 23720, GammaStar = 40, Sco25 = 2.8, Ha_Sco = -24460, alpha = 0.3, theta = 0.7, PAR = 1000, T = 25).

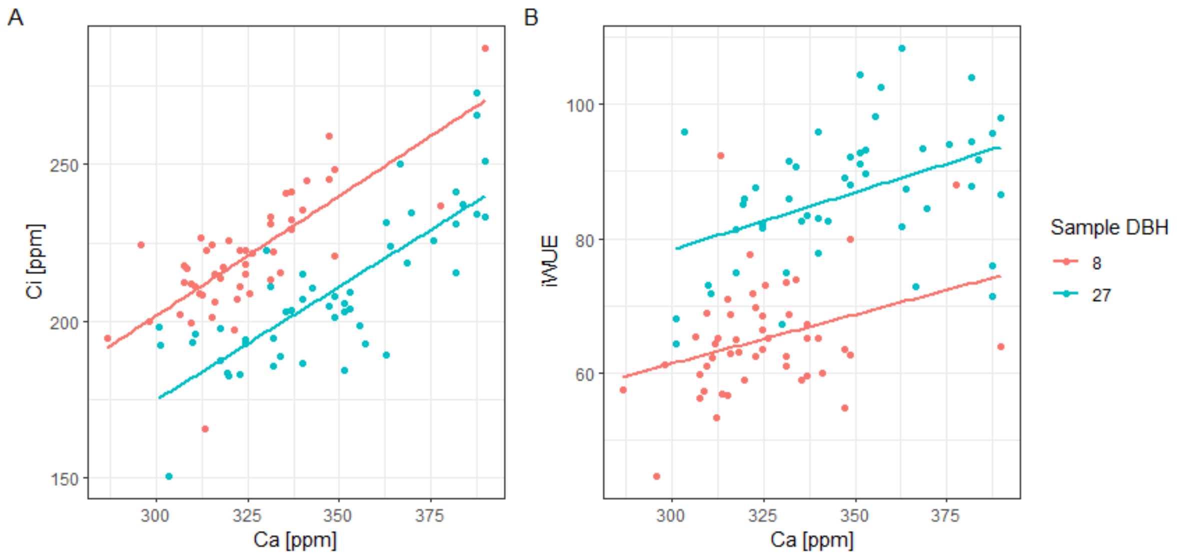

Figure S15. Leaf internal CO_2 (C_i) and intrinsic water use efficiency (iWUE) estimates based on ^{13}C isotopes for *T. ciliata* in our site in Thailand. The relationship of C_i and C_a for trees with a DBH of 8 cm and 27 cm, data from Sleen et al 2015 (A). Measurements are based on five rings pooled around the two different diameters. It is clear from the intercept of lines in A that C_i is consistently lower for trees with a larger diameter, but the response to C_a is similar in strength. Lower C_i due to tree size increases iWUE, while higher C_i due to higher C_a also increases iWUE (B).

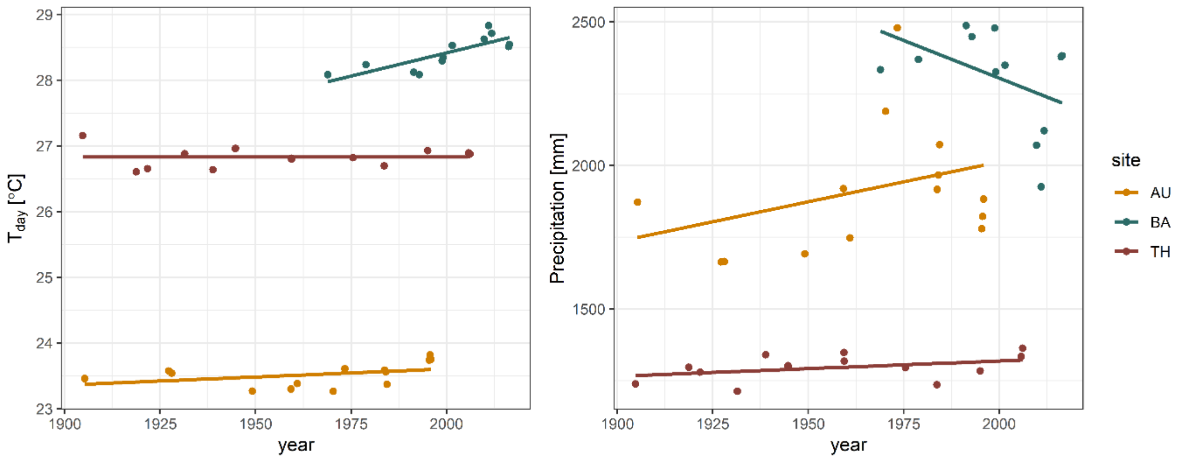

Figure S16. Trends in climatic variables as included in our data over time. Lines are based on the ggplot2 smoothing function, with method linear.

References

Arel-Bundock V. 2022. modelsummary: Data and Model Summaries in R. *Journal of statistical software* **103**: 1-23.

Betson TR, Augusti A, Schleucher J. 2006. Quantification of deuterium isotopomers of tree-ring cellulose using nuclear magnetic resonance. *Analytical Chemistry* **78**(24): 8406-8411.

Daniel Lüdecke, Mattan S. Ben-Shachar, Indrajeet Patil, Brenton M. Wiernik, Etienne Bacher, Rémi Thériault, Makowski D 2022. easystats: Framework for Easy Statistical Modeling, Visualization, and Reporting. CRAN.

Ehlers I, Augusti A, Betson TR, Nilsson MB, Marshall JD, Schleucher J. 2015. Detecting long-term metabolic shifts using isotopomers: CO_2 -driven suppression of photorespiration in C_3 plants over the 20th century. *Proceedings of the National Academy of Sciences of the United States of America* **112**(51): 15585-15590.

Farquhar GD, Von Caemmerer S, Berry JA. 1980. A biochemical model of photosynthetic CO_2 assimilation in leaves of C_3 species. *Planta* **149**(1): 78-90.

Schleucher J, Vanderveer P, Markley JL, Sharkey TD. 1999. Intramolecular deuterium distributions reveal disequilibrium of chloroplast phosphoglucose isomerase. *Plant, Cell & Environment* **22**(5): 525-533.
